# Tuning the selectivity of NH_3_ oxidation via cooperative electronic interactions between platinum and copper sites

**DOI:** 10.1038/s41467-024-54820-y

**Published:** 2025-01-02

**Authors:** Lu Chen, Xuze Guan, Zhaofu Fei, Hiroyuki Asakura, Lun Zhang, Zhipeng Wang, Xinlian Su, Zhangyi Yao, Luke L. Keenan, Shusaku Hayama, Matthijs A. van Spronsen, Burcu Karagoz, Georg Held, Christopher S. Allen, David G. Hopkinson, Donato Decarolis, June Callison, Paul J. Dyson, Feng Ryan Wang

**Affiliations:** 1 Department of Chemical Engineering, University College London, London, WC1E 7JE UK; 2 Institute of Chemical Sciences and Engineering, École Polytechnique Fedérale de Lausanne (EPFL), 1015 Lausanne, Switzerland; 3 Department of Applied Chemistry, Faculty of Science and Engineering, Kindai University, Higashi-Osaka, Osaka, 577-8502 Japan; 4 Diamond Light Source Ltd., Harwell Science and Innovation Campus, Chilton, Didcot, OX11 0DE UK; 5 electron Physical Science Imaging Center, Diamond Light Source Ltd., Harwell Science and Innovation Campus, Chilton, Didcot, OX11 0DE UK; 6 Department of Materials, University of Oxford, Oxford, OX1 3PH UK; 7 UK Catalysis Hub, Research Complex at Harwell (RCaH), Rutherford Appleton Laboratory, Harwell, OX11 0FA UK

**Keywords:** Heterogeneous catalysis, Catalytic mechanisms, Catalyst synthesis

## Abstract

Selective catalytic oxidation (SCO) of NH_3_ to N_2_ is one of the most effective methods used to eliminate NH_3_ emissions. However, achieving high conversion over a wide operating temperature range while avoiding over-oxidation to NO_x_ remains a significant challenge. Here, we report a bi-metallic surficial catalyst (Pt_S_CuO/Al_2_O_3_) with improved Pt atom efficiency that overcomes the limitations of current catalysts. It achieves full NH_3_ conversion at 250 °C with a weight hourly space velocity of 600 ml NH_3_·h^−1^·g^−1^, which is 50 °C lower than commercial Pt/Al_2_O_3_, and maintains high N_2_ selectivity through a wide temperature window. *Operando* XAFS studies reveal that the surface Pt atoms in Pt_S_CuO/Al_2_O_3_ enhance the redox properties of the Cu species, thus accelerating the Cu^2+^ reduction rate and improving the rate of the NH_3_-SCO reaction. Moreover, a synergistic effect between Pt and Cu sites in Pt_S_CuO/Al_2_O_3_ contributes to the high selectivity by facilitating internal selective catalytic reduction.

## Abstract (web summary)

Selective catalytic oxidation (SCO) of NH_3_ to N_2_ is a highly effective approach for reducing NH_3_ emissions, though achieving high conversion across a broad temperature range without over-oxidation to NOx remains challenging. Here, the authors introduce a bi-metallic surficial Pt-Cu catalyst that effectively removes NH_3_ from both stationary and mobile exhaust sources via SCO.

## Introduction

The global emissions of ammonia (NH_3_) from vehicle exhaust and industrial waste gas streams are estimated to exceed 220,000 tonnes per year, presenting a severe environmental threat and impacting on human health^1,2^. With the utilization of NH_3_ as a fuel for various modes of transportation, emissions might increase considerably^3,4^. Consequently, selective catalytic oxidation (SCO) of NH_3_ to nitrogen N_2_ (avoiding over-oxidation to NO_x_) is increasingly imperative in order to address the issue of unreacted NH_3_ emissions (referred to as NH_3_ slip). NH_3_-SCO offers an attractive approach for treating gas flows containing 100−5000 ppm NH_3_ and abundant O_2_, such as waste gases from chemical manufacturing processes, selective catalytic reduction (SCR) of NO_x_ units, biomass gasification processes and NH_3_ combustion turbines.

NH_3_-SCO catalysts must be capable of achieving complete conversion of NH_3_ to N_2_, while avoiding overoxidation to NO_x_, and maintaining high stability over a broad operating temperature range (100 °C < T < 450 °C). Noble metal-based catalysts, such as commercial Pt/Al_2_O_3_, are renowned for their high efficiencies, however, their selectivity to N_2_ is low, (ca. 50% for commercial Pt/Al_2_O_3_) and the atomic efficiency is also low^5^. First row transition metals such as Cu and Fe exhibit high N_2_ selectivity, but require higher operating temperatures (300–500 °C)^6,7^. In order to enhance the N_2_ selectivity of Pt-based catalysts, recent developments have aimed at the development of catalysts that leverage the strengths of noble metals and non-noble transition metals through bifunctional catalyst design. Efforts have focused on the bi-metallic catalysts that form somewhat random alloys or mixed oxides^8–16^, and the location of noble metal atoms is difficult to control. However, noble metals nanoparticles favour the oxidation of ammonia to NO, whereas non-noble transition metal oxides are able to selectively reduce NO with NH_3_ to generate N_2_^7–9^. Therefore, by combining the two types of catalysts using a rational design approach, i.e. ensuring the ratio of the Pt and CuO is optimized to the relative reaction rates of both processes, it should be possible to obtain a highly active and selective catalyst for the NH_3_-SCO reaction.

Herein, by precisely controlling the amount of Pt atoms on the surface (S) of CuO nanoparticles (NPs, Pt_S_CuO/Al_2_O_3_), the catalytic activity and N_2_ selectivity were enhanced, surpassing commercial Pt/Al_2_O_3_ catalysts and also standard co-reduced bi-metallic Pt_N_CuO/Al_2_O_3_ (N stands for normal) catalysts. Based on *operando* and time-resolved X-ray absorption fine structure (XAFS) studies, the Pt atoms are active in the oxidation of NH_3_. Moreover, the Pt atoms also accelerate the redox activity of the Cu species facilitating the NH_3_-SCO reaction to achieve high activity and high selectivity to N_2_.

## Results and Discussion

### Synthesis and structural characterisation of the Pt_S_CuO/Al_2_O_3_ catalyst

**Fig. 1:**
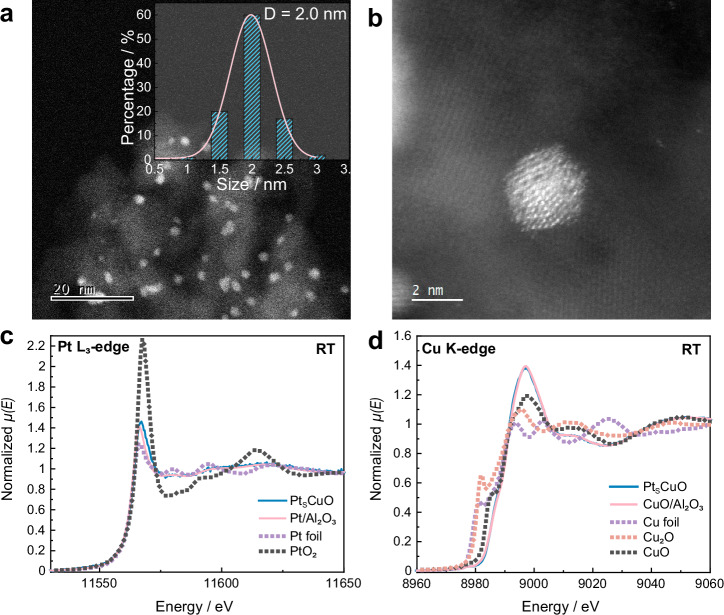
Characterisation of Pt_S_CuO/Al_2_O_3_ and Pt_N_CuO/Al_2_O_3_. **a**, **b** TEM images of Pt_S_CuO/Al_2_O_3_ (the inset in **a** shows the particle size distribution. The average particle size was calculated based on more than 100 particles.); Pt L_3_-edge and Cu K-edge EXAFS of Pt_S_CuO/Al_2_O_3_ (**c**) and Pt_N_CuO/Al_2_O_3_ (**d**).

The surface bi-metallic Pt_S_CuO/Al_2_O_3_ catalyst was synthesized using galvanic replacement. As the galvanic replacement between Pt and Cu atoms is initiated on the CuO NPs, the Pt atoms form a thin shell on the NP surfaces. For comparison, normal alloy Pt_N_CuO/Al_2_O_3_ with the same chemical compositions was synthesized by wet impregnation, resulting in randomly distributed Pt atoms within the CuO NPs. The NPs in both Pt_S_CuO/Al_2_O_3_ and Pt_N_CuO/Al_2_O_3_ have a similar average particle size of ca. 2 nm (Fig. 1a, b, Supplementary Figs. S1 and S2). The Energy-dispersive spectrometry (EDS) map of Pt_S_CuO/Al_2_O_3_ displays a uniform elemental distribution over a wide area (Fig. S3), and within a single Pt_S_CuO/Al_2_O_3_ particle (Fig. S4). X-ray diffraction patterns of Pt_S_CuO/Al_2_O_3_ and Pt_N_CuO/Al_2_O_3_ shows no obvious distinctions from the Al_2_O_3_ support (PDF #10-0425) (Fig. S5), indicative of small NPs. Fine-scanned X-ray photoelectron spectroscopy (XPS) of Pt_S_CuO/Al_2_O_3_ and Pt_N_CuO/Al_2_O_3_ reveals Cu 2*p*_3/2_ peaks at 931.8 and 934.6 eV corresponding to Cu^+^ and Cu^2+^, respectively (Fig. S6). The chemical environments of the copper ions in both Pt_S_CuO/Al_2_O_3_ and Pt_N_CuO/Al_2_O_3_ are similar, as determined from extended X-ray absorption fine structure (EXAFS) and X-ray absorption near-edge structure (XANES) measurements, but differ in the chemical states of Pt (Fig. 1c, d and S7). The fitting of EXAFS data reveals the differences between the structures of Pt_S_CuO/Al_2_O_3_ and Pt_N_CuO/Al_2_O_3_ (Table S1). Pt_S_CuO/Al_2_O_3_ has a larger Pt-Pt coordination number (C.N.) of 5.8 ± 1.5 and smaller Pt-Cu C.N. of 1.1 ± 0.7 compared to Pt_N_CuO/Al_2_O_3_, which has a Pt-Pt C.N. of 4.5 ± 1.4 and a Pt-Cu C.N. of 5.8 ± 1.7. A smaller overall coordination indicates that most Pt atoms in the Pt_S_CuO/Al_2_O_3_ are on the surface whereas the Pt atoms in Pt_N_CuO/Al_2_O_3_ reside on the surface and in the NP bulk.

### Evaluation of the catalysts in the NH_3_-SCO reaction

**Fig. 2:**
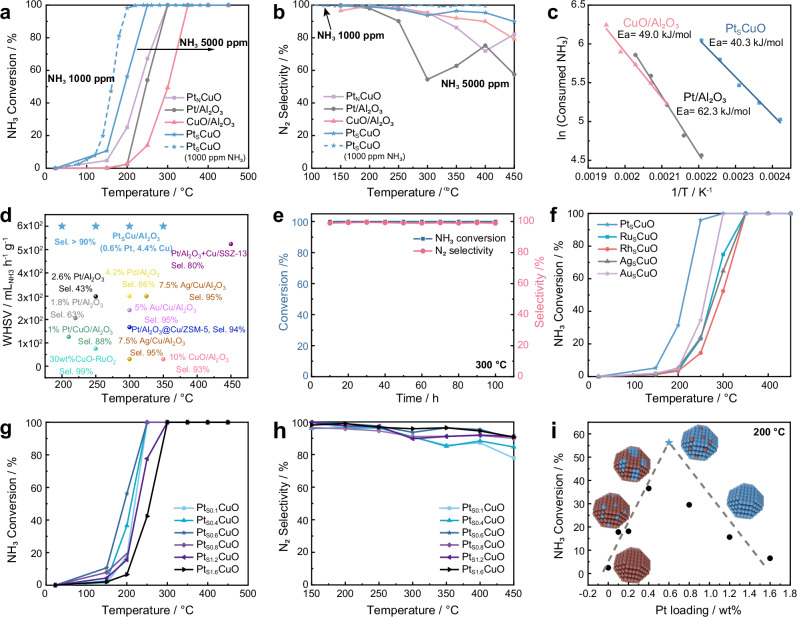
Evaluation of Pt_S_CuO/Al_2_O_3_ in comparison to other catalysts in the NH_3_-SCO reaction. **a, b** NH_3_ conversion and N_2_ selectivity as a function of temperature; **c** activation energy of Pt_S_CuO/Al_2_O_3_, CuO/Al_2_O_3_ Pt/Al_2_O_3_; **d** WSHV with refs (Table S2^10–23^); **e** stability test of Pt_S_CuO/Al_2_O_3_ at 200 °C; **f** activity of M_S_CuO/Al_2_O_3_ catalysts (M = Pt, Ru, Rh, Ag, or Au); **g**, **h** NH_3_ conversion and N_2_ selectivity as a function of Pt loading and temperature; **i** NH_3_ conversion of Pt_S_CuO/Al_2_O_3_ with different Pt loadings at 200 °C. Reaction conditions: 50 mg catalyst, 5000 ppm NH_3_, 5% O_2_ balanced in He, gas flow: 100 mL/min, WHSV = 600 mL NH_3_·h^−1^·g^−1^.

The activities of Pt_S_CuO/Al_2_O_3_, Pt_N_CuO/Al_2_O_3_, CuO/Al_2_O_3_ and Pt/Al_2_O_3_ were evaluated in the NH_3_-SCO reaction. Remarkably, at 200 °C, Pt_S_CuO/Al_2_O_3_ consisting of 0.6 wt% Pt and 4.4 % Cu, exhibits a 30-fold higher activity than the commercial catalyst Pt/Al_2_O_3_ containing 1 wt% Pt. Moreover, Pt_S_CuO/Al_2_O_3_ also shows the highest activity with the lowest T_50_ (T at 50% conversion), and complete NH_3_ conversion was achieved at around 250 °C (Fig. 2a, S8 and S9). Under realistic NH_3_ slip conditions (1000 ppm NH_3_, weight hourly space velocity (WHSV) of 120 ml_NH3_·h^−1^·g^−1^), full conversion could be achieved at 200 °C using the Pt_S_CuO/Al_2_O_3_ catalyst (Fig. 2a). Notably, Pt_N_CuO/Al_2_O_3_ with identical Pt and Cu loadings to Pt_S_CuO/Al_2_O_3_, requires 300 °C to reach the full conversion under equivalent conditions (Fig. 2a). When the temperature is higher than 250 °C, the N_2_ selectivity of the Pt_N_CuO/Al_2_O_3_, CuO/Al_2_O_3_ and Pt/Al_2_O_3_ catalysts decrease, due to an increase in the rate of ammonia oxidation to NO (Step 1: 4 NH_3_ + 5O_2_ → 4 NO + 6 H_2_O). In comparison, Pt_S_CuO/Al_2_O_3_ consistently maintains >90% selectivity to N_2_ even at full conversion (Fig. 2b). Pt/Al_2_O_3_ has the lowest selectivity to N_2_ at temperatures above 250 °C, e.g. 55% selectively at 300 °C.

A series of control experiments were carried out to ensure the influence of internal and external diffusion could be excluded from the kinetic experiments (Fig. S10a, b). The apparent activation energy of Pt_S_CuO/Al_2_O_3_ is 40 kJ/mol, which is lower than that of CuO/Al_2_O_3_ and Pt/Al_2_O_3_ (Fig. 2c), suggesting that the superior activity in the NH_3_-SCO reaction is due to interactions between the surface Pt atoms and underlying Cu atoms (note that other metal-Cu combinations are less efficient, see below). Additionally, Pt_S_CuO/Al_2_O_3_ outperforms most previously reported catalysts in terms of selectivity to N_2_ (Fig. 2d and Table S2)^10–23^. Compared to benchmark Pt- and Cu-based catalysts, such as the Pt/Al_2_O_3_@Cu/ZSM‑5 core−shell catalyst and Pt/Al_2_O_3_ + Cu/SSZ−13 dual-layer wash-coated monolith catalyst, Pt_S_CuO/Al_2_O_3_ displays a higher activity at low temperatures and maintains high selectivity at high temperatures^20–23^. Notably, Pt_S_CuO/Al_2_O_3_ also exhibits exceptional stability, without any signs of reduced activity and N_2_ selectivity even after 100 h of continuous operation at 200 or 300 °C (Fig. 2e and S10c). After the reaction, the size distribution of the NPs in the Pt_S_CuO/Al_2_O_3_ catalyst remains unchanged (Fig. S11), affirming its remarkable stability. The Pt_S_CuO/Al_2_O_3_ catalyst is able to operate at low temperatures (200 °C) that are well suited to the cold start of a vehicle, and its high N_2_ selectivity over a wide operating temperature range (150–450 °C) makes it practical for removal of NH_3_ from diesel exhausts.

As mentioned above, the Pt and Cu combination in Pt_S_CuO/Al_2_O_3_ shows the highest activity, with M_S_CuO/Al_2_O_3_ (M = Pt, Ru, Rh, Ag, or Au) catalysts prepared via galvanic replacement being less active (Fig. 2f and S12). To investigate the special effect of Pt coverage on CuO NPs in the NH_3_-SCO reaction, the Pt loading was systematically varied from 0 to 1.6 wt% and the performance of the catalysts was evaluated (Fig. 2g–i and S13). Despite all catalysts exhibiting similar particle size distributions irrespective of the Pt loading (Figs. S14−16), their catalytic performance differs significantly. At 200 °C the activity of Pt_S_CuO/Al_2_O_3_ shows a volcano plot-like trend with 0.6 wt% Pt, i.e. Pt_S0.6_CuO/Al_2_O_3_, being the most active, indicating that optimal Pt surface coverage is important (Fig. 2i). Hence, the Pt-Pt and Pt-Cu coordination environments play a vital role in the NH_3_-SCO reaction.

### Formation of Cu^+^ and Pt^0^ at steady state

The internal selective catalytic reduction (i-SCR) mechanism is widely accepted with copper-based catalysts^18,24–27^, in which ammonia is oxidized to NO_x_, and then the formed NO_x_ species react further with NH_3_ on the Cu sites to afford N_2_ (Step 2). The first step is the conversion of NH_3_ and is usually the rate-determining step in NH3 oxidation, which involves metal redox processes^27^.

Step 1: 4 NH_3_ + 5O_2_ → 4 NO + 6 H_2_O

Step 2: 4 NO + 4 NH_3_ + O_2_ → 4 N_2_ + 6 H_2_O

4 NO + 4 NH_3_ + 3 O_2_ → 4 N_2_O + 6 H_2_O

**Fig. 3:**
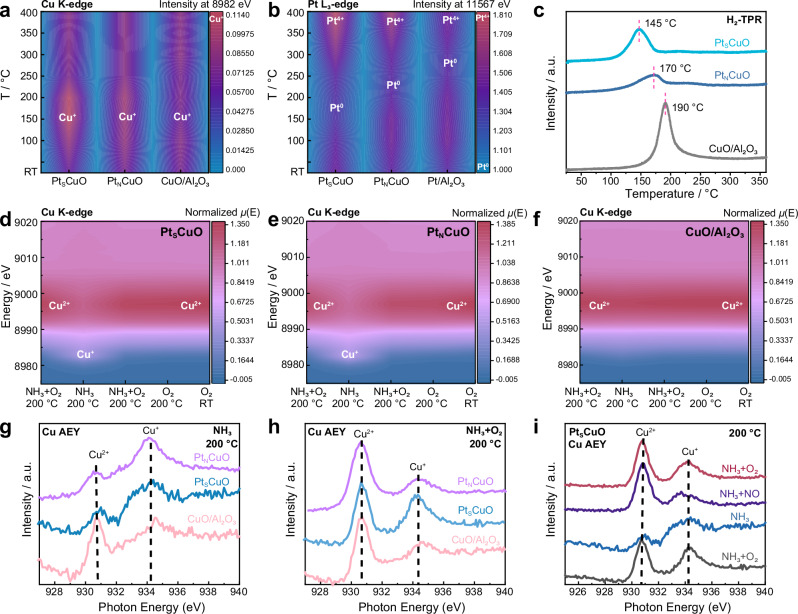
*Operando* studies on the redox behaviour of Cu and Pt in different catalysts. **a** *operando* Cu K-edge XAFS, signal intensity of the Cu^+^ 1s-4p transition peak at 8982 eV in a NH_3_/O_2_ atmosphere at different temperatures; **b** *operando* Pt L_3_-edge, signal intensity of the white line of the Pt L_3_-edge at 11567 eV in a NH_3_/O_2_ atmosphere at different temperatures (reaction conditions: 5000 ppm NH_3_, 5% O_2_ balanced in He, gas flow: 100 mL/min); **c** H_2_-TPR of different catalysts; *operando* Cu K-edge XANES spectra of Pt_S_Cu/Al_2_O_3_ (**d**), Pt_N_Cu/Al_2_O_3_ (**e**) and CuO/Al_2_O_3_ (**f**) in different gases at 200 °C; in situ NAP-NEXAFS spectra, Cu L-edge (AEY mode) of different catalysts at 200 °C under NH_3_ (**g**) or NH_3_/O_2_ (**h**) atmospheres; **i** Cu L-edge (AEY mode) of Pt_S_Cu/Al_2_O_3_ under various gas atmospheres at 200 °C (gas pressure 1 mbar).

**Fig. 4:**
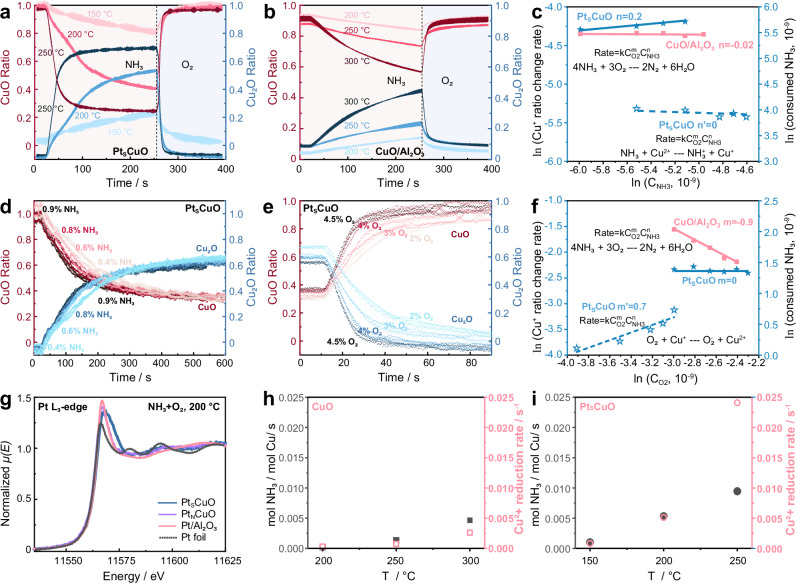
Kinetic studies and adsorption behaviours of the Pt_S_CuO/Al_2_O_3_ and CuO/Al_2_O_3_ catalysts. Change of CuO:Cu_2_O ratio in Pt_S_CuO/Al_2_O_3_ (**a**) and CuO/Al_2_O_3_ (**b**) with time under NH_3_ or O_2_ environments at different temperatures (the catalysts are exposed to a flow of 5000 ppm NH_3_/He or 5% O_2_/He with flow rate 15 mL/min); change of CuO:Cu_2_O ratio in the Pt_S_CuO/Al_2_O_3_ catalyst with time under NH_3_ (**d**) and O_2_ (**e**) environments at different gas concentrations; reaction order for NH_3_ (**c**) and O_2_ (**f**) in the whole apparent reaction or half reaction; **g** *operando* Cu K-edge XANES spectra of the Pt_S_Cu/Al_2_O_3_, Pt_N_Cu/Al_2_O_3_ and CuO/Al_2_O_3_ catalysts under NH_3_ + O_2_ at 200 °C (5000 ppm NH_3_, 5% O_2_ balanced in He, gas flow: 100 mL/min); Comparation between TOF of the full NH_3_-SCO reaction and Cu^2+^ reduction rate in Cu/Al_2_O_3_ (**h**) and Pt_S_Cu/Al_2_O_3_ (**i**).

Adding Pt into CuO will boost Step 1, as metallic Pt is usually the best catalyst for low-temperature combustion reactions^1,28,29^. In *operando* XAFS under SCO conditions, indicated that the initial Pt^4+^ species in the Pt_S_CuO/Al_2_O_3_, Pt_N_CuO/Al_2_O_3_ and Pt/Al_2_O_3_ catalysts are reduced to Pt^0^ in the temperature ranges 100–250, 200–300 and 250–300 °C, respectively, and are subsequently re-oxidized to Pt^4+^ (Fig. 3b and S17–19). Pt/γ-Al_2_O_3_ (2 wt%) has been previously studied by operando XAS and was shown to undergo reduction between 230 − 260 °C^23^, which is similar the catalysts reported herein. When the temperature is lower than 250 °C, the Pt species are mainly in metallic state in Pt_S_CuO/Al_2_O_3_, which improved the activity of Step 1 (Figs. 3b, 4g), while Pt species are in a more oxidated state in Pt_N_CuO/Al_2_O_3_, and Pt/Al_2_O_3_. In situ, XAFS under different gas atmospheres confirms that Pt species in Pt_S_CuO/Al_2_O_3_ are readily oxidized when the feed gas composition changes from reducing to oxidizing at 200 °C (Fig. S20).

Although Pt is predominant in the surface layer, the underlying Cu atoms modulate the surface electronic structure to improve both the catalytic activity and durability. To assess the redox behaviour (essential for the formation of N_2_ from the oxidation of NH_3_) and monitor structural changes of the CuO NPs and surface Pt under relevant NH_3_-SCO reaction conditions, *operando* XAFS measurements were performed in fluorescence mode at the Pt L_3_-edge and transmission mode at the Cu K-edge under steady-state conditions between 25 and 400 °C (Fig. 3a–c and S21–23). Figure 3a illustrates the evolution of the Cu K-edge XAFS spectra at the Cu^+^ 1 s → 4p transition in Pt_S_CuO/Al_2_O_3_, Pt_N_CuO/Al_2_O_3_, and CuO/Al_2_O_3_ during the NH_3_-SCO reaction (NH_3_ 5000 ppm, O_2_ 5%) as a function of temperature. Between 100 and 250 °C, Cu^2+^ in Pt_S_CuO/Al_2_O_3_ is partially reduced to Cu^+^, even under excess O_2_, as evidenced by the more pronounced peak intensity of the Cu K-edge at 8982 eV (the typical feature for Cu^I^(NH_3_)_2_ 1 s → 4p XANES) (Fig. 3a). Above 250 ^o^C the Cu^+^ feature at 8982 eV disappears and is accompanied by an increase in the white-line intensity at 8996 eV (Fig. 3a, S21 and 24), indicative of oxidation of Cu^+^ to Cu^2+^. In comparison, the peak intensity of the Cu K-edge at 8982 eV in Pt_N_CuO/Al_2_O_3_ only increases slightly between 100 and 250 °C, whereas Cu^+^ in CuO/Al_2_O_3_ is almost constant over the entire temperature range (Fig. 3a, S23–25). As the feed gas composition is changed from reducing to oxidizing, the Cu species in Pt_S_CuO/Al_2_O_3_ are readily reduced and oxidized at 200 °C (Fig. 3d and S26), whereas the Cu species in CuO/Al_2_O_3_ do not change (Fig. 3f).

In situ near ambient pressure-near edge X-ray absorption fine structures (NAP-NEXAFS) of the catalysts under a NH_3_ atmosphere (1 mbar) confirms the superior redox properties of Pt_S_CuO/Al_2_O_3_, with a more pronounced peak observed at 934.2 eV corresponding to Cu^+^ (Fig. 3g). Additionally, the formation of Cu^+^ in Pt_S_CuO/Al_2_O_3_ under a NH_3_/O_2_ atmosphere was validated by in situ NAP-NEXAFS (Fig. 3h). In the NAP-NEXAFS studies, the Cu species in the Pt_S_CuO/Al_2_O_3_ catalyst return to their original redox states after exposure to different gases (Fig. 3i). This switching between Cu^+^ and Cu^2+^ confirms the reversibility of the oxidation state of Cu species in Pt_S_Cu/Al_2_O_3_, as well as the high stability of the catalyst together with the 100 h stability tests at 200 °C (Fig. 2e). Based on the *operando* XAFS and in situ NAP-NEXAFS studies, the Cu species in Pt_S_CuO/Al_2_O_3_ undergo facile redox switching compared to Pt_N_CuO/Al_2_O_3_ and CuO/Al_2_O_3_, which is in agreement with the hydrogen-temperature programmed reduction (H_2_-TPR) results, in which the Pt_S_CuO/Al_2_O_3_ catalyst has the lowest reduction temperature, indicating that surface Pt species enhance the reduction ability of CuO NPs (Fig. 3c).

Reduction of Cu^2+^ to Cu^+^ and Pt^4+^ to Pt^0^ takes place in the same temperature range for Pt_S_CuO/Al_2_O_3_, which is not the case for Pt_N_CuO/Al_2_O_3_, indicating a better redox synergy between the two metals in the former. Hence, it would appear that Pt^0^ induces the reduction of Cu^2+^ to Cu^+^ in Pt_S_CuO/Al_2_O_3_. Pt_N_CuO/Al_2_O_3_ and Pt/Al_2_O_3_ are more resistant to reduction and oxidation in the course of the reaction, since both the Cu and Pt oxidation states do not vary significantly (Fig. 3a, b). In the light-off curve of Pt_S_Cu/Al_2_O_3_ shown in Fig. S27, the reaction rate increases rapidly from 150 °C, which corresponds to the formation temperature of the Cu^I^(NH_3_)_2_ species (Fig. 3a), so that the formation of the Cu^+^ is considered to be a trigger for the NH_3_-SCO reaction. The highest activity for the NH_3_-SCO reaction between 100 and 250 °C is found for Pt_S_CuO/Al_2_O_3_, which has the highest abundance of Cu^+^. Compared with Pt_N_CuO/Al_2_O_3_ and CuO/Al_2_O_3_, the superior redox properties of Cu and Pt species in Pt_S_CuO/Al_2_O_3_ lead to higher activity at lower temperatures.

### Determination of the Cu^+/2+^ redox rate

In situ energy dispersive EXAFS (EDE) under modulation excitation of net-reducing (NH_3_) to net-oxidizing (O_2_) gas environments was used to probe the redox behaviour of Cu^+/2+^ in the Pt_S_CuO/Al_2_O_3_ and CuO/Al_2_O_3_ catalysts. For both catalysts oxidation to Cu^2+^ is much faster than reduction to Cu^+^, partially due to the higher concentration of O_2_ (5%) used compared to NH_3_ (5000 ppm) (Fig. 4a, b). Notably, the rates for oxidation and reduction in Pt_S_CuO/Al_2_O_3_ is much higher than in CuO/Al_2_O_3_ at all temperatures, which is in agreement with the steady-state study (see Fig. 3).

At 200 °C, the reaction order of NH_3_ in the half-reaction (2NH_3_ + 6CuO → N_2_ + 3Cu_2_O + 3H_2_O) is facilitated by Pt is almost 0, which is consistent with the apparent reaction order of NH_3_ (0.2) in the overall SCO reaction (4NH_3_ + 3O_2_ → 2N_2_ + 6H_2_O) (Fig. 4c, d). In this case, the NH_3_ that reacts is limited to those that absorb on the Pt_S_CuO/Al_2_O_3_ surface, a key step for both the SCO reaction and the reduction of Cu^2+^. The amount of Cu^+^ in Pt_S_CuO/Al_2_O_3_ is dependent on the oxygen concentration (Fig. 4e, f), with a reaction order of 0.7. The O_2_ order for the SCO reaction is 0 and −0.9 for the Pt_S_CuO/Al_2_O_3_ and CuO/Al_2_O_3_ catalysts (Fig. 4f), respectively, indicating that the lattice O participates in the oxidation process due to the surface coverage of NH_3_ on the Pt_S_CuO/Al_2_O_3_ catalyst (Mars–van Krevelen mechanism)^30,31^. The lower reaction order for O_2_ in the NH_3_-SCO reaction implies facile oxygen activation takes place on the surface of the Pt_S_CuO/Al_2_O_3_ catalyst. In the absence of NH_3_, however, for the reaction O_2_ + 2 Cu_2_O → 4 CuO, the gas phase oxygen surface coverage is rate determining^27^. Overall, the redox behaviour of Cu^+/2+^ is important for the i-SCR mechanism in the overall NH_3_-SCO process^12,32^, with the Cu^+/2+^ redox kinetics aligned with the overall SCO kinetics.

To further validate that the Cu^2+^ reduction is the rate-determining step, we compare the Cu^+/2+^ redox rate with the turnover frequency (TOF) of the whole NH_3_-SCO reaction (Fig. 4h, i, S28). With both Pt_S_CuO/Al_2_O_3_ and CuO/Al_2_O_3_, the Cu^+^ oxidation rate is faster than Cu^2+^ reduction rate, and the Cu^+^ oxidation rate consistently surpasses the NH_3_ oxidation rate (i.e. TOF of the whole NH_3_-SCO reaction) (Fig. S28). This implies that the Cu^+^ oxidation step is not the rate-determining step in both Pt_S_CuO/Al_2_O_3_ and CuO/Al_2_O_3_ catalysts. For both catalysts, the TOF is comparable to the rate of Cu^2+^ reduction to Cu^+^, indicating that Cu^2+^ reduction should be the rate-determining step (Fig. 4h, i). At 250 °C, NH_3_ conversion for Pt_S_CuO/Al_2_O_3_ is over 50% and thus in the diffusion region. Therefore, the Cu^2+^ reduction rate from the in situ EDE experiment is much higher than the calculated TOF from converion (Fig. 4i). It is noteworthy that the Cu^+/2+^ redox rate with Pt_S_CuO/Al_2_O_3_ is consistently superior than the Cu^+/2+^ redox rate with CuO/Al_2_O_3_ across all temperatures. This confirms that the enhanced Cu^+/2+^ redox rate promotes the activity.

### Fast Cu^+/2+^ redox rate leads to higher selectivity of N_2_

Pt/Al_2_O_3_ is effective at oxidizing ammonia but lacks selectivity to nitrogen gas, especially above 250 °C (Fig. 2b and S9). With Pt/Al_2_O_3_, unfavourable over-oxidation to N_2_O and NO takes place on the surface of the PtO_2_ nanoparticles (Step 2 in the i-SCR)^11,19,33^. Compared to Pt/Al_2_O_3_, the Cu in the Pt_S_CuO/Al_2_O_3_ catalyst considerably improves the selectivity to N_2_ while not affecting the low-temperature activity (Fig. 2a, b), due to the excellent SCR performance of Cu species^34,35^. In Step 2 of the i-SCR mechanism, NO oxidation on the Cu^2+^ site forms HONO-like species, and the Cu^2+^ is reduced to Cu^+^^36^. Subsequently, the HONO-like species react further with adsorbed NH_3_ to generate N_2_ and H_2_O, and the Cu^+^ is then oxidized by O_2_ to regenerate Cu^2+^, thus completing the redox cycle.

**Fig. 5:**
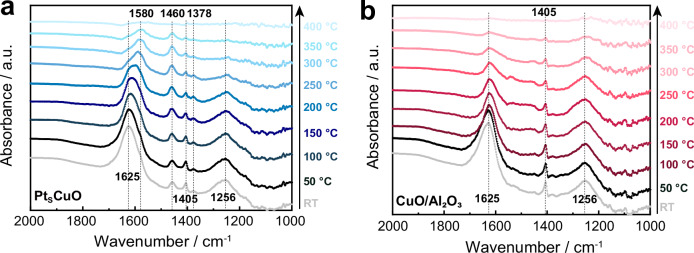
*Operando* DRIFTS studies. *Operando* DRIFTS spectra of Pt_S_Cu/Al_2_O_3_ (**a**) and Cu/Al_2_O_3_ (**b**) as a function of temperature (the catalysts were exposed to a flow of 5000 ppm NH_3_ and 5% O_2_ for 20 min at different temperatures).

*Operando* DRIFTS confirm that the combination of Pt and Cu sites in Pt_S_CuO/Al_2_O_3_ contributes to improved selectivity via the i-SCR (Fig. 5a, b), to achieve the high selectivity to N_2_ (above 90% over all temperatures)^37^. The bands observed at 1625 and 1,256 cm^−1^ may be assigned to asymmetric and symmetric deformation of ammonia chemisorbed on Lewis acid sites of Al_2_O_3_, respectively^27^. Large amounts of NH_3_ (1625 and 1256 cm^−1^) are adsorbed on the Lewis acid sites at temperatures below 200 °C. The amount of adsorbed NH_3_ decreases as the temperature increases, and –NH_2_ species (evidenced by a peak at 1580 cm^–1^ in the IR spectra) gradually emerge at temperatures above 250 °C^38,39^. It is likely that NH_3_ dissociatively adsorbs as −NH_2_ on the surface of the Pt_S_CuO/Al_2_O_3_ catalysts. It has been reported that NH_3_ adsorption on Lewis acid sites is responsible for NH_3_ oxidation activity, but does not significantly impact N_2_ selectivity^40^. The presence of Pt in the Pt_S_CuO/Al_2_O_3_ catalyst enhances the Cu^+^/Cu^2+^ redox recycle, which leads to a higher NH_3_ oxidation rate. The Lewis acid sites on Al_2_O_3_ observed at 1625 and 1256 cm^−1^ have more pronounced peaks and are more reactive than the Brønsted acid sites (1460 cm^−1^) in the NH_3_-SCO reaction, since the intensity of the peak at 1460 cm^−1^ hardly changes. It has also been reported that NH_3_ species adsorbed on Brønsted acid sites can promote the conversion of NO_x_ formed during NH_3_ oxidation, thereby improving overall selectivity to N_2_ through the i-SCR mechanism^41^. NH_3_ adsorbed on Brønsted acid sites were detected from room temperature to 350 °C on the Pt_S_CuO/Al_2_O_3_ catalyst, but are not observed on the CuO/Al_2_O_3_ catalyst. This phenomenon suggests that NH_3_ species adsorbed on Brønsted acid sites of the Pt_S_CuO/Al_2_O_3_ catalyst are more stable than those on CuO/Al_2_O_3_. Presumably the increased stability significantly enhances the selectivity of the NH_3_–SCO reaction to N_2_ on the Pt_S_CuO/Al_2_O_3_ catalyst. The peak at 1405 cm^−1^ may be assigned to NH_3_ coordinated to Cu and the peak at 1378 cm^−1^ may be attributed to NH_3_ coordinated to Pt^36,42^. The presence of Pt in the Pt_S_CuO/Al_2_O_3_ catalyst boosts the Cu^+^/Cu^2+^ redox cycle, which leads to a higher i-SCR rate and therefore a higher yield to N_2_.

NH_3_ emissions are expected to increase in the future and selective catalytic oxidation (SCO) of NH_3_ to N_2_ is one of the principle approaches used to eliminate these emissions. The Pt_S_CuO/Al_2_O_3_ catalyst reported here is superior to the commercial Pt/Al_2_O_3_ catalysts with respect to both activity and selectivity, achieving full conversion of 5000 ppm NH_3_ at 250 °C, with selectivity to N_2_ between 90 and 100% depending upon the temperature. Based on the moderate operating conditions and the excellent selectivity across a broad temperature range, the catalyst is even suitable for cold start applications such as controlling diesel engine emissions. *Operando* XAFS and time-resolved energy dispersive EXAFS studies were used to show that the enhanced redox rate of the Cu species, induced by the Pt sites in Pt_S_CuO/Al_2_O_3_, leads to enhanced activity. *Operando* DRIFTS further demonstrate that the interactions between the Pt and Cu sites contribute to the high selectivity via the i-SCR reaction. Overall, this study illustrates the dynamic changes in the chemical state of the active sites in nanoscale catalysts under relevant reaction conditions, highlighting the importance of *operando* studies to gain a mechanistic understanding of structure–reactivity correlations. The results will help to guide future catalyst design, such as non-noble metal counterparts.

## Methods

### Catalyst preparation

#### Synthesis of Cu/Al_2_O_3_

γ-Al_2_O_3_ (0.5 g, Johnson Matthey) was dispersed in ethanol (20 mL) with vigorous stirring at room temperature. To the resulting suspension, a solution containing Cu(NO_3_)_2_^.^3H_2_O (100 mg, 0.4 mmol) in ethanol (5 mL) was slowly added, and stirring was continued for 12 h at room temperature. The reaction mixture was then heated at 40 °C under stirring until all the solvent had evaporated. The remaining solid was heated to 300 °C for 1 h under 15% H_2_/Ar at a heating rate of 5 °C/min in a tube furnace to afford Cu/Al_2_O_3_.

#### Synthesis of Pt_S_Cu/Al_2_O_3_

The Pt_S_Cu/Al_2_O_3_ catalyst was prepared via galvanic replacement between the Cu NPs and H_2_PtCl_6_. Cu/Al_2_O_3_ was dispersed in ethanol (25 mL) under a nitrogen atmosphere, and the resulting suspension was heated at 60 °C for 10 min. A solution of H_2_PtCl_6_ (6 mg) dissolved in ethanol (2 mL) was added slowly to the reaction mixture. After stirring for 6 h at 60 °C, the solution was cooled to room temperature, and then the solid was collected by centrifugation and washed with ethanol (5 × 30 mL). After drying at 40 °C for 24 h, the Pt_S_Cu/Al_2_O_3_ catalyst was obtained as a grey powder.

#### Synthesis of Pt/Al_2_O_3_

1 wt% Pt/Al_2_O_3_ was prepared using the wetness impregnation method. H_2_PtCl_6_·6H_2_O (13.4 mg) was dissolved in deionised water and then added into γ-Al_2_O_3_ (0.5 g) ethanol suspension. The solvent was then removed at 60 °C and the sample was heated at 300 °C for 1 h under 15% H_2_/Ar at a heating rate of 5 °C/min.

### Ex situ characterisation

#### X-ray diffraction (XRD)

XRD patterns were recorded on a StadiP diffractometer (STOE) with a Mo source (Kα = 0.7093165 Å). The operating voltage and current were 40 kV and 30 mA, respectively. 2θ in the range of 2–40° were collected with a resolution of 0.015° for each step.

#### H_2_-TPR

the measurements were performed on an FD-2000 reactor and quantified using an AO2000 analyser. Typically, 50 mg catalyst was placed in a quartz tube and pre-treated in He at 300 °C for 30 min to remove the surface absorbed species. After cooling to room temperature, the sample was heated in 5% H_2_/N_2_ (100 mL/min) at a rate of 5 °C/min, then kept at 350 °C for 30 mins.

#### TEM

Aberration-corrected bright field (BF) and annular dark field (ADF) scanning transmission electron microscopy (STEM) was performed on a JEOL (Tokyo, Japan) ARM300CF (E02) operating 300 kV. Simultaneous energy dispersive x-ray (EDX) spectroscopy and aberration-corrected BF/ADF-STEM imaging was performed on a JEOL ARM200CF (E01) operating at 200 kV and equipped with JEOL dual silicon drift detectors at the electron Physical Sciences Imaging Centre (ePSIC) at Diamond Light Source (UK) (DLS). The ARM300CF operated with a convergence semi-angle of 26.2 mrad and BF and ADF collection semi-angles of 0-31.6 and 77.0-209.4, respectively. The ARM200CF operated with a convergence semi-angle of 23.0 mrad with BF and ADF collection semi-angles of 0–21.9 and 37.5–128.3 respectively. Single-pass EDX spectra were collected with drift correction. Data were acquired and processed using the Gatan Microscopy Suite (a.k.a. Digital Micrograph)^43^. Nanoscale catalyst particles were prepared via a standard preparation route: a small amount (<20 mg) of catalyst powder was dispersed in approximately 5 ml of ethanol, before sonication and drop casting approximately 1 ml of supernatant onto holey carbon coated, gold TEM support grids. Gold was used instead of the more typical copper grid to avoid overlapping fluorescent signals with the sample during EDX mapping. The average particle size was calculated based on more than 100 particles for each sample.

#### X-ray absorption fine structure (XAFS)

XAFS of the Pt L_3_-edge (11.564 keV) and Cu K-edge (8.979 keV) were carried out at the Diamond Light Source (UK) and SPring-8 (Japan). Samples were directly pressed into pellets for fluorescence measurements of the Pt L_3_-edge and transmission measurements of the Cu K-edge. Pt foil or Cu foil standards were used for energy shift calibration.

XAFS data involved merging three spectra to improve signal quality and were processed using the Demeter software package (including Athena and Artemis). Athena software was used to analyse the XANES data. Artemis software was used to fit the *k*^2^-weighted EXAFS data in real space with 3.0 Å^−1^ < *k* < 12.0 Å^−1^ and 1.0 Å <*R* < 3.3 Å. The calculated amplitude reduction factor S_0_^2^ from the EXAFS analysis of Cu foil was 0.878, which was used as a fixed parameter for EXAFS fitting. The coordination numbers and bond lengths were calculated based on the reported structures from the Crystal open database: Cu (No. 9013014), CuO (No. 1011148), Pt (No. 9008480), and PtO_2_ (No. 1008935).

### *Operando* Pt L_3_-edge and Cu K-edge XAFS

*Operando* XAFS experiments were performed at SPring-8 (Japan). 100 mg of pelletised catalysts were measured at 8780–10200 eV for Cu K edge in transmission mode and 11345–12745 eV for Pt L_3_-edge in fluorescence mode at different temperatures and under various gas atmospheres. Spectra processing was performed with Athena software.

### *Operando* DRIFTS

DRIFTS was performed on a PerkinElmer Frontier FT-IR Spectrometer. The sample was heated in He at 350 °C for 30 min to remove surface contamination. After cooling to room temperature, the sample was exposed to 5000 ppm NH_3_ or 5%O_2_/He for 30 minutes, during which spectra were recorded. Then, the sample was heated from 30 to 450 °C with a ramp of 10 °C/min. The spectra were recorded from 4400 to 500 cm^−1^ with a resolution of 2 cm^−1^. Background spectra were recorded in He and subtracted from the sample spectrum for each measurement.

### In situ energy dispersive EXAFS (EDE)

Cu K edge EDE measurements were carried out on the I20-EDE beamline at the Diamond Light Source (UK). For in situ experiments the samples were sieved (125–200 µm) and filled into a quartz tube (5.5 mm). An identical tube filled with Al_2_O_3_ was used as the background. The gas flow was 40 mL/min and spectra were taken when switching the gases. Each condition was run 6 times, and the results were merged for the final spectra.

### In situ near ambient pressure-near edge X-ray absorption fine structure (NAP-NEXAFS) spectroscopy

In situ NAP-NEXAFS experiments were performed on the B07 beamline at the Diamond Light Source (UK)^44^. The X-rays are sourced from a bending magnet (D41) and a plane grating monochromator (PGM) with an energy range from 80 to 2000 eV (soft X-ray range) and flux of 6 × 10^10^ photons/s with 0.1 A ring current using a 111 µm slit and an 80 µm × 200 µm beam spot size. The reaction products were monitored online using an electron impact mass spectrometer (“PRISMA”, PFEIFFER VACUUM GmbH, Asslar (Germany)) connected directly to the main experimental chamber by a leak valve. The pressure in the specimen chamber was precisely controlled at 1 mbar by simultaneous operation of several mass flow controllers for reactive gases and a PID-controlled throttle valve for pumping gas out. Temperature control was achieved by two K-type thermocouples. NEXAFS spectra at Cu L-edge (925–940 eV) were measured in Auger electron yield (AEY) mode.

Measurements were performed under various gas conditions with a total pressure of 1 mbar. In situ experiments employing Pt_S_Cu/Al_2_O_3_, Pt_N_Cu/Al_2_O_3_ and CuO/Al_2_O_3_ were carried out at 200 °C under NH_3_ + O_2_ (NH_3_/O_2_: 1:10) or NH_3_. For experiments carried out under various gas atmospheres at 200 °C, the sequence of different gas atmospheres follows NH_3_ + O_2_ (ratio: 1:10), NH_3_, NO + NH_3_ (ratio: 1:1) and NH_3_ + O_2_ (ratio: 1:10). All spectra were recorded under steady-state conditions.

### Catalytic performance measurements

The performance of the catalysts in the NH_3_-SCO reaction was evaluated in a fixed-bed flow reactor at a gas flow rate of 100 mL/min, which consists of 5000 ppm NH_3_, 5 vol% O_2_, and the He balance. In a typical experiment, 50 mg of catalyst was placed in the reaction tube, and quantification of the products was performed with an online quadrupole mass spectrometer quantitative gas analyser (Hiden Analytical, UK). The reaction was investigated at temperatures ranging from 100 °C to 450 °C. The reaction was kept stable for at least 30 minutes after attaining a steady state at each reaction temperature to detect the MS signals of (NH_3_ and O_2_) and products (N_2_, N_2_O and NO).

To obtain the activation energy, 50 mg catalyst powder was immobilized in a fixed-bed flow reactor and a gas flow rate of 100 mL/min, consisting of 5000 ppm NH_3_, 5 vol% O_2_, and a He balance was applied. The reaction was investigated at temperatures ranging from 140–180 °C for Pt_S_Cu/Al_2_O_3_, 180–220 °C for Pt/Al_2_O_3_ and 200–240 °C for CuO/Al_2_O_3_.

To obtain the reaction order for NH_3_, the O_2_ was kept at 5%, while the concentration of NH_3_ was varied, i.e. 2500, 4000, 5000, 6000 and 7000 ppm. All the tests were performed at 150 °C for Pt_S_Cu/Al_2_O_3_, and 220 °C for CuO/Al_2_O_3_.

To obtain the reaction order for O_2_, the NH_3_ was kept at 5000 ppm, while the concentration of O_2_ was varied, i.e. 5, 6, 7, 8, 9 and 10%. All the tests were performed at 150 °C for Pt_S_Cu/Al_2_O_3_ and 220 °C for CuO/Al_2_O_3_.

To evaluate the external diffusion limitation, catalyst performance was tested under different flow rates. Different amounts of catalyst powder was immobilized in a fixed-bed flow reactor and the WHSV maintained at 600 mL NH_3_/g/h. The reaction was investigated at different flow rates of 25, 50, 75, 100, 125 or 150 mL/min at 200 °C and 250 °C.

To evaluate the internal diffusion limitation, the performance of catalysts of differing particle sizes was tested. 50 mg catalyst powder with different particle sizes, including <250 µm, 250–500 µm, and >500 µm, were immobilized in a fixed-bed flow reactor and a gas flow rate of 100 mL/min, consisting of 5000 ppm NH_3_, 5 vol% O_2_, and a He balance was applied.

## Supplementary information

Supplementary Information

Transparent Peer Review file

## Data Availability

All data generated in this study are provided in the article and Supplementary Information files.
