# Supplementary material for: Tuning the selectivity of NH3 oxidation via cooperative electronic interactions between platinum and copper sites
Source: Nat Commun. 2025 Jan 2;16:26. doi: 10.1038/s41467-024-54820-y (PMC11697224; doi:10.1038/s41467-024-54820-y)
Supplement: Supplementary file 1 — Supplementary Information [file 41467_2024_54820_MOESM1_ESM.pdf]

Supplementary Information

Tuning the selectivity of NH_3 oxidation via cooperative electronic interactions between platinum and copper sites

Lu Chen[1,†,*], Xuze Guan[1,†], Zhaofu Fei[2], Hiroyuki Asakura[3], Lun Zhang[1], Zhipeng Wang[1], Xinlian Su[1], Zhangyi Yao[1], Luke Keenan[4], Shusaku Hayama[4], Matthijs A. Van Spronsen[4], Burcu Karagoz[4], Georg Held[4], Christopher S. Allen[5,6], David G. Hopkinson[5], Donato Decarolis[4,7], June Callison[7], Paul J. Dyson[2,*], Feng Ryan Wang[1,*]

1. Department of Chemical Engineering, University College London, London *WC1E 7JE*, United Kingdom
2. Institute of Chemical Sciences and Engineering, École Polytechnique Fedérale de Lausanne (EPFL), 1015 Lausanne, Switzerland
3. Department of Applied Chemistry, Faculty of Science and Engineering, Kindai University, Higashi-Osaka, Osaka, 577-8502, Japan
4. Diamond Light Source Ltd., Harwell Science and Innovation Campus, Chilton, Didcot OX11 0DE, United Kingdom
5. electron Physical Science Imaging Center, Diamond Light Source Ltd., Harwell Science and Innovation Campus, Chilton, Didcot OX11 0DE, United Kingdom
6. Department of Materials, University of Oxford, OX1 3PH, United Kingdom
7. UK Catalysis Hub, Research Complex at Harwell (RCaH), Rutherford Appleton Laboratory, Harwell OX11 0FA, United Kingdom

†These authors contributed equally to this work.

*e-mail: lc962@cam.ac.uk, paul.dyson@epfl.ch, ryan.wang@ucl.ac.uk

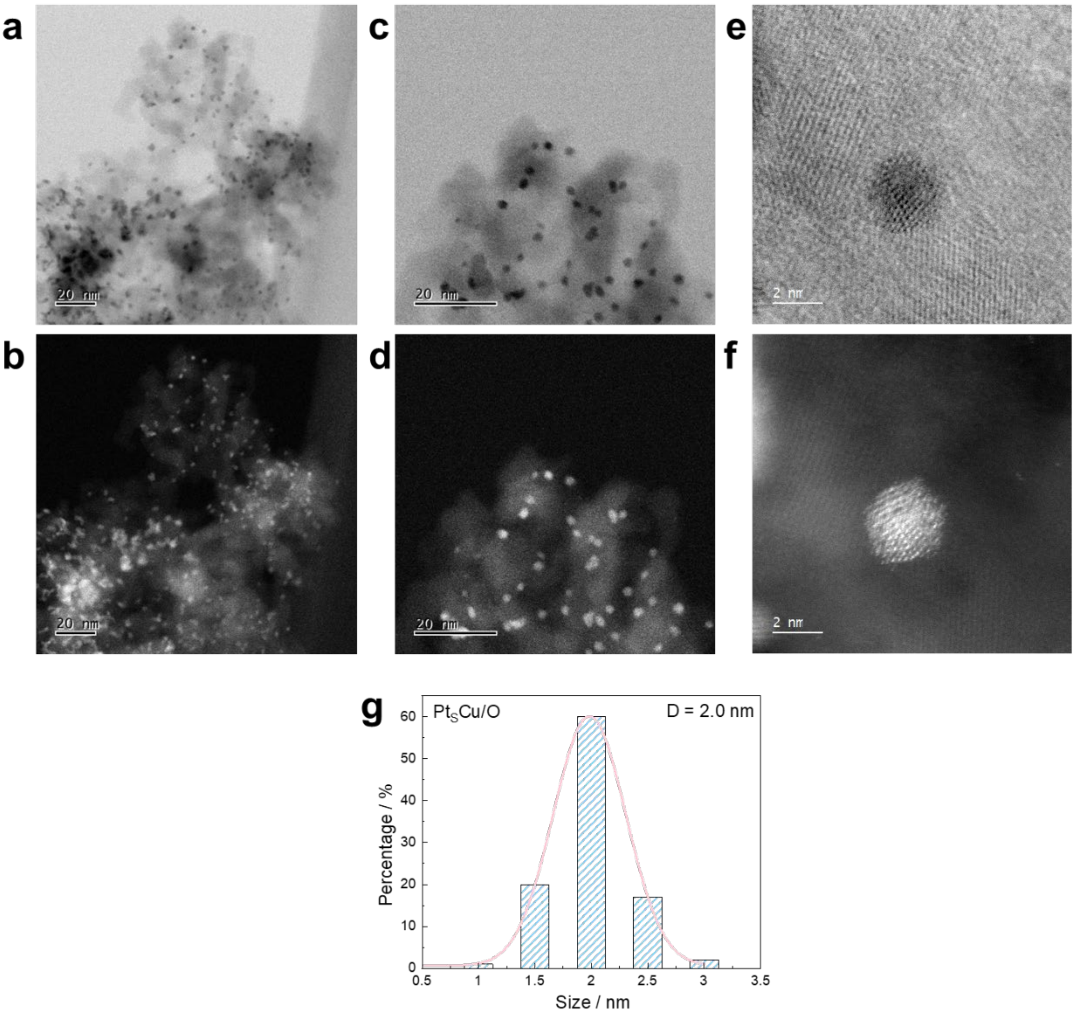

Figure S1. STEM images and particle size distribution of Pt_SCuO/Al_2O_3 before reaction. BF-STEM (**a, c, e**) and HAADF-STEM (**b, d, f**) images of Pt_SCuO/Al_2O_3 before reaction at different magnifications and the particle size distribution (**g**). The average particle size was calculated based on more than 100 particles.

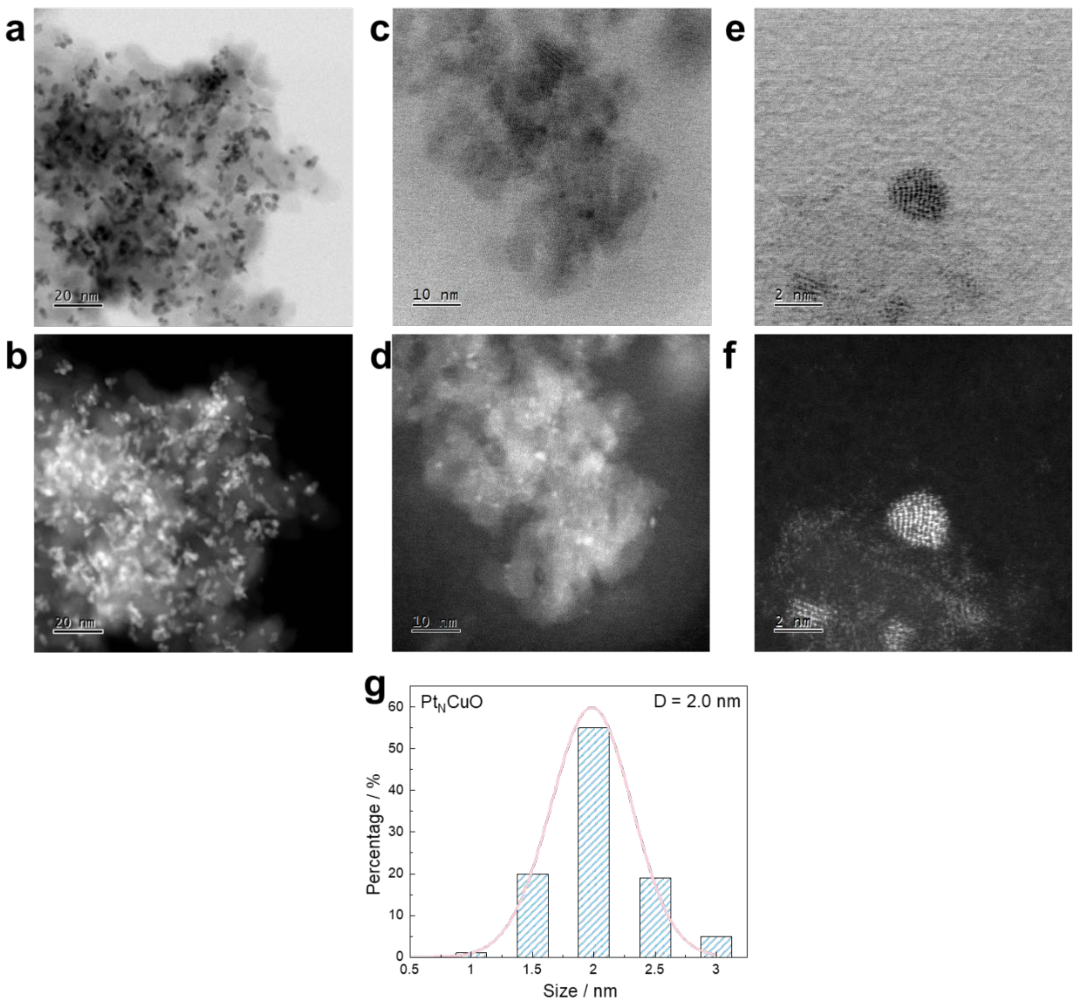

Figure S2. STEM images and particle size distribution of Pt_NCuO/Al_2O_3 before reaction. BF-STEM (**a, c, e**) and HAADF-STEM (**b, d, f**) images of Pt_NCuO/Al_2O_3 before reaction at different magnifications and the particle size distribution (**g**). The average particle size was calculated based on more than 100 particles.

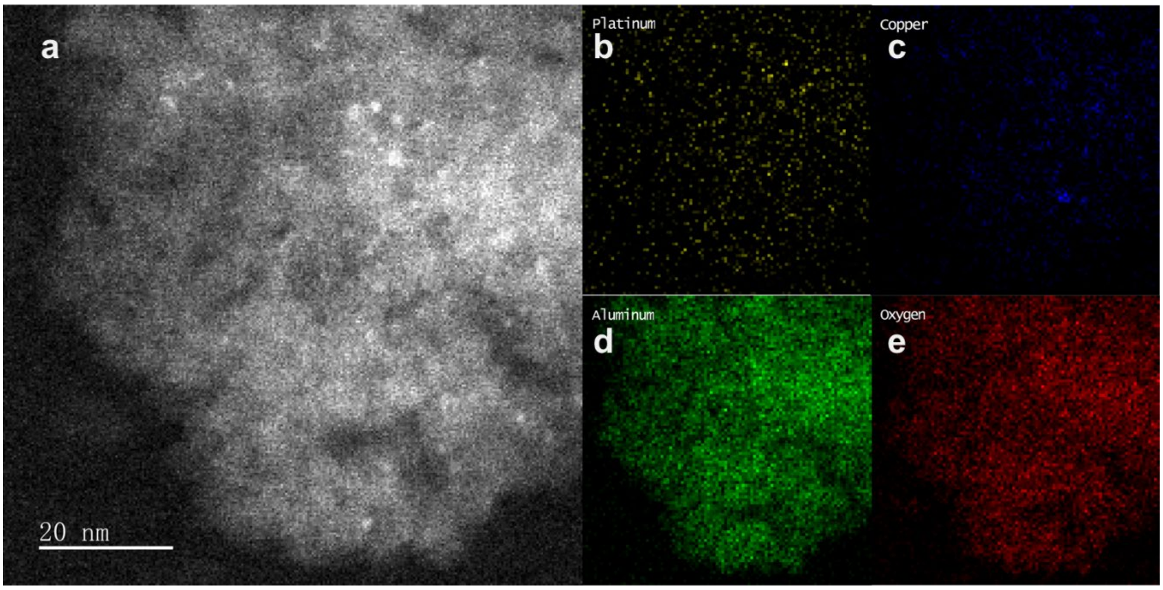

Figure S3. Compositional elemental mapping of Pt_5CuO/Al_2O_3. a STEM image of Pt_5CuO/Al_2O_3. **b-e** EDS elemental mapping of Pt_5CuO/Al_2O_3.

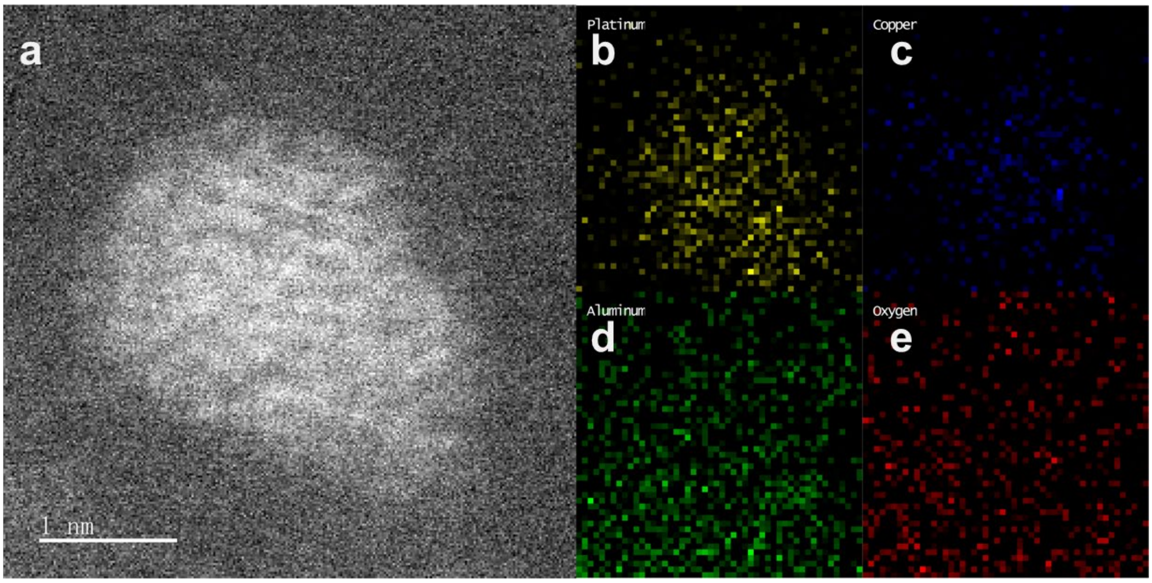

Figure S4. Compositional elemental mapping of a single nanoparticle in Pt_5CuO/Al_2O_3. **a** STEM image of a single nanoparticle in Pt_5CuO/Al_2O_3. **b-e** EDS elemental mapping of a single nanoparticle in Pt_5CuO/Al_2O_3.

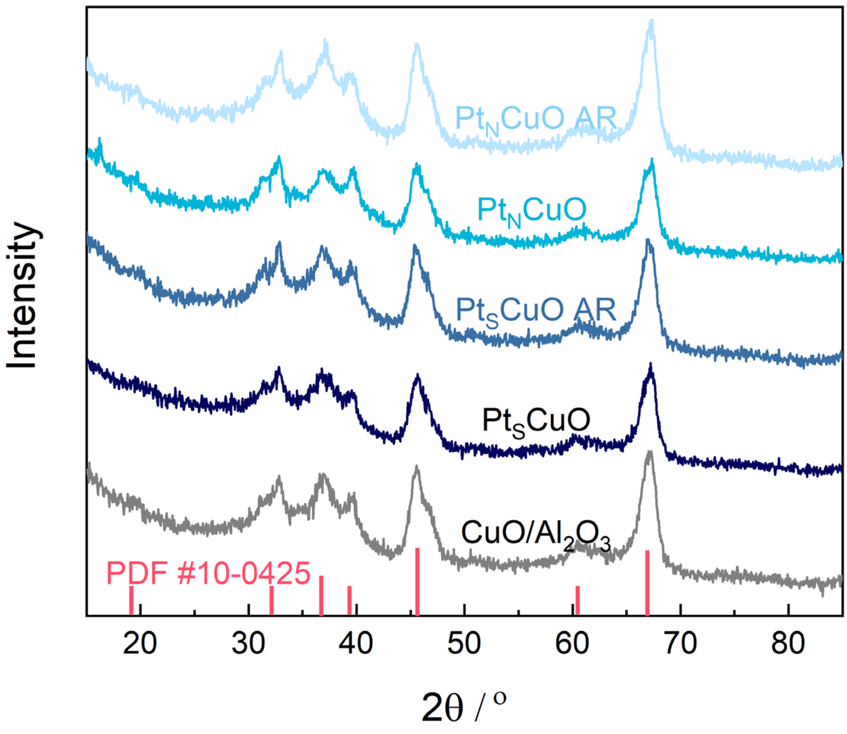

Figure S5. X-ray diffraction (XRD) patterns of Pt_SCuO/Al_2O_3, Pt_NCuO/Al_2O_3 and CuO/Al_2O_3 before and after reaction. Al_2O_3 PDF #10-0425 is provided at the bottom in pink. (AR: after reaction)

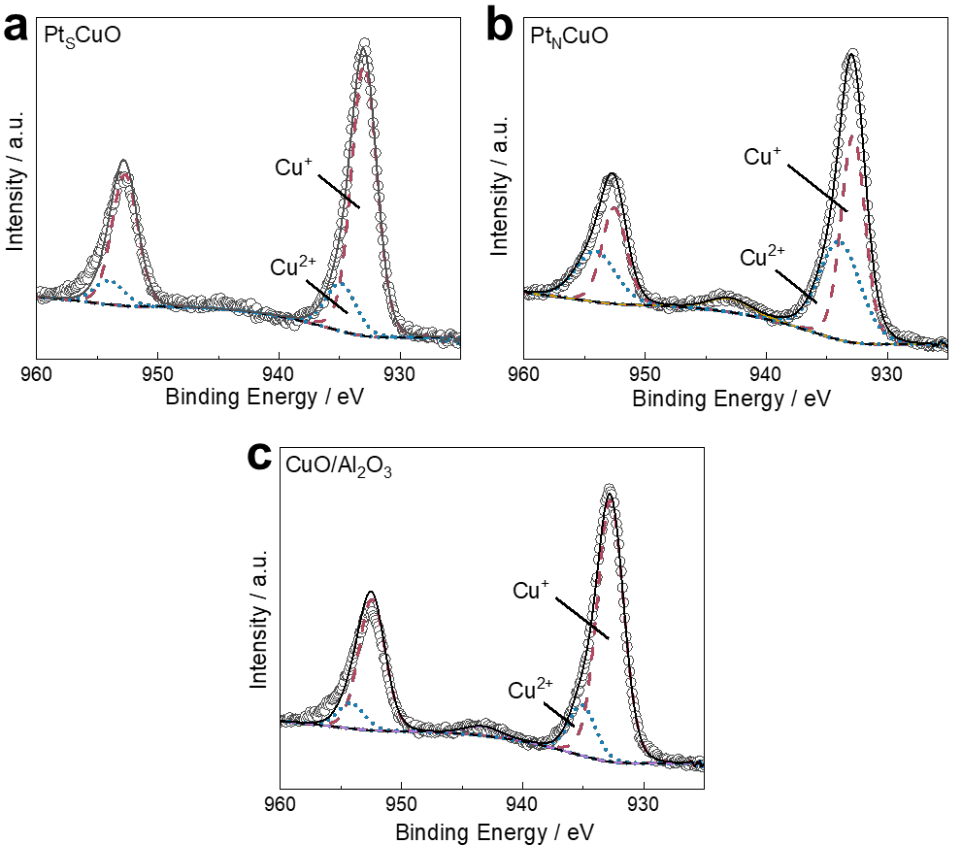

Figure S6. Cu 2*p* XPS spectra of different catalysts. Cu 2*p* XPS spectra of Pt_SCuO/Al_2O_3 (**a**), Pt_NCuO/Al_2O_3 (**b**) and CuO/Al_2O_3 (**c**). Pink lines correspond to Cu^+ 2*p*, and blue lines correspond to Cu^{2+} 2*p*.

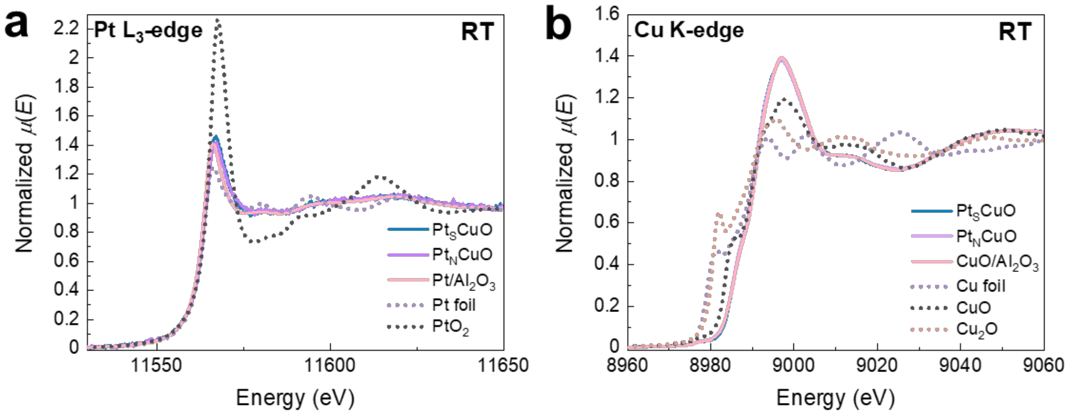

Figure S7. EXAFS spectra of different catalysts. Pt L_3-edge (**a**) and Cu K-edge (**b**) EXAFS spectra of Pt_SCuO/Al_2O_3, Pt_NCuO/Al_2O_3 and CuO/Al_2O_3.

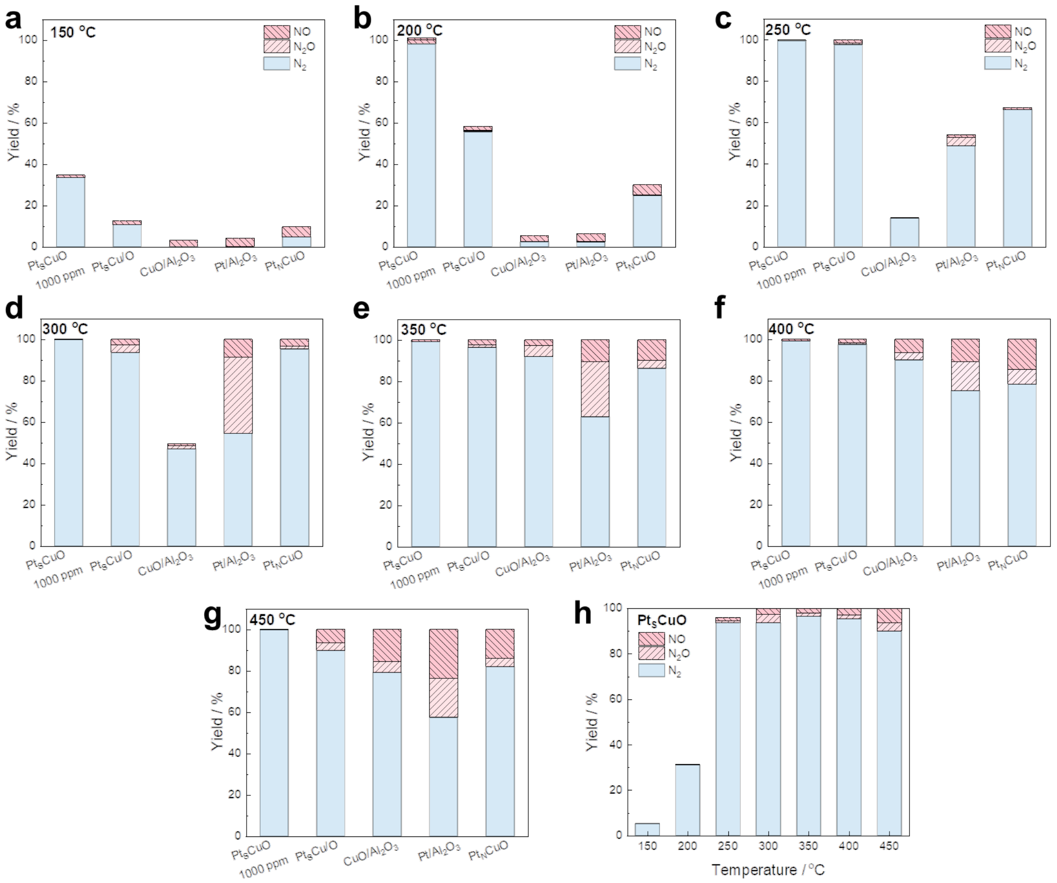

Figure S8. Performance of different catalysts in the oxidation of NH_3 at different temperatures. a 150 °C, **b** 200 °C, **c** 250 °C, **d** 300 °C, **e** 350 °C, **f** 400 °C, **g** 450 °C, **h** Pt_SCuO/Al_2O_3 (reaction conditions: 50 mg catalyst, 5000 ppm NH_3, 5% O_2 balanced in He, gas flow: 100 mL/min, WHSV=600 mL $NH_3 \cdot h^{-1} \cdot g^{-1}$).

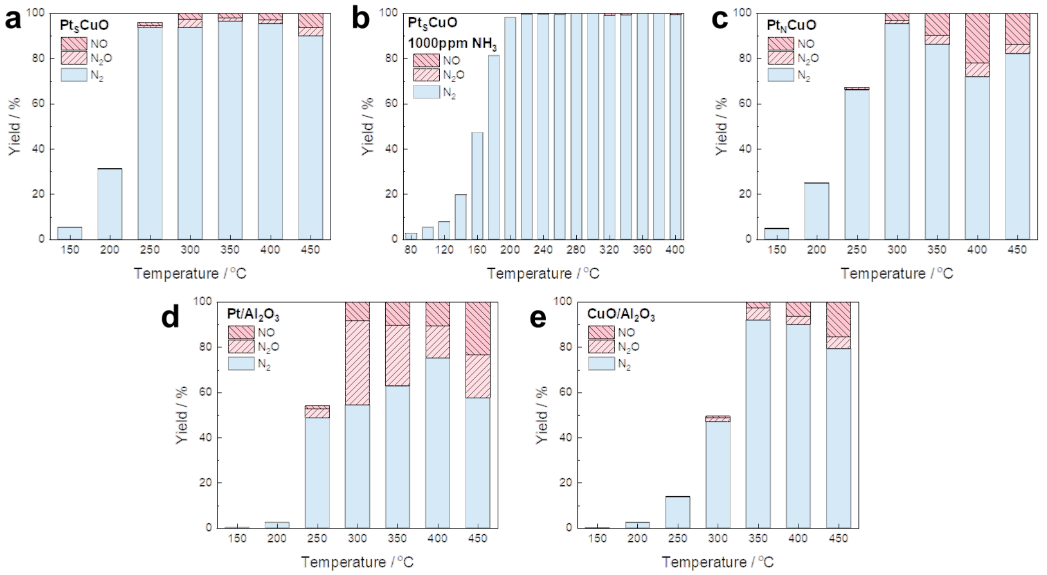

Figure S9. Catalytic performance of different catalysts in NH_3 oxidation. a Pt_SCuO/Al_2O_3, **b** Pt_SCuO/Al_2O_3 at 1000 ppm NH_3, **c** Pt_NCuO/Al_2O_3, **d** Pt/Al_2O_3, **e** CuO/Al_2O_3 (reaction condition: 50 mg catalyst, 5000 ppm NH_3, 5% O_2 balanced in He, gas flow: 100 mL/min, WHSV=600 mL $NH_3 \cdot h^{-1} \cdot g^{-1}$).

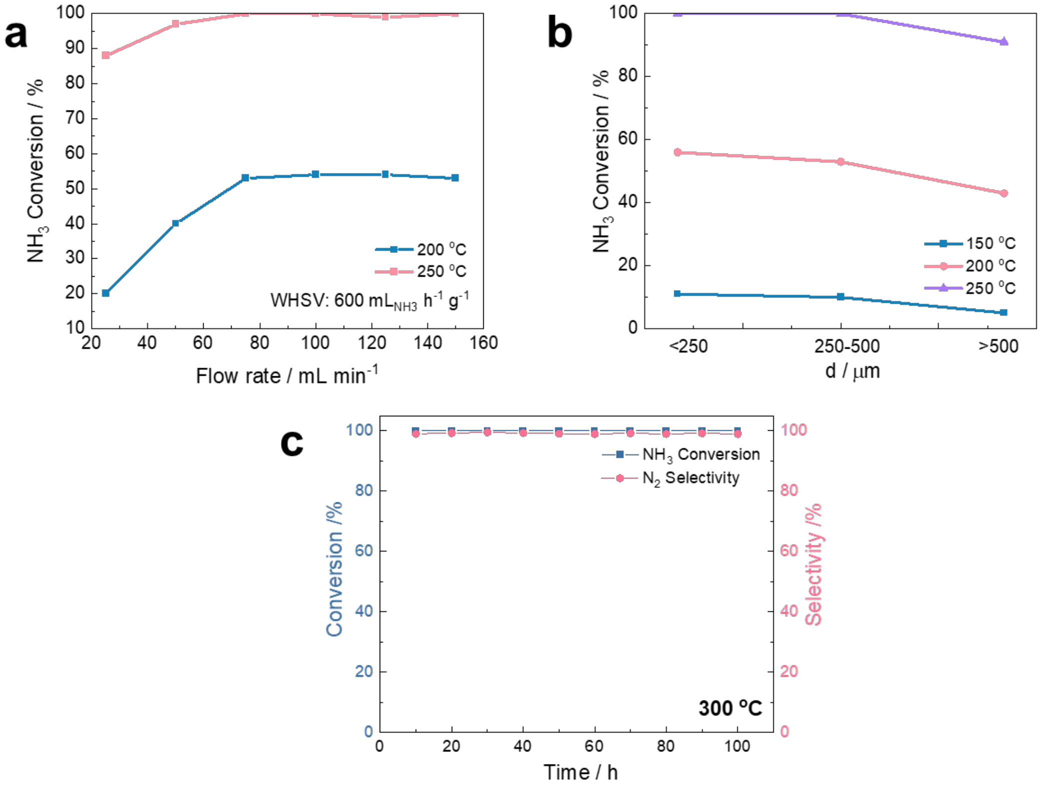

Figure S10. Influence of diffusion limitations and stability tests. a Influence of flow rate on NH_3 conversion; **b** Influence of particle size on NH_3 conversion; **c** Stability of Pt_5CuO/Al_2O_3 at 300 °C.

To rule out external diffusion limitations, experiments were conducted using different amounts of catalyst at the same WHSV. When the flow rate exceeds 75 mL/min, no external diffusion is present. Since the flow rate used in our experiments are larger than this threshold, there is no external diffusion limitation under the reaction conditions used. Additionally, the catalytic performance with catalysts of differing particle sizes was tested to evaluate potential internal diffusion limitations (Figure S8b). When the particle size is < 500 μm, internal diffusion limitations do not impact on the catalytic performance. In our experiment, the catalyst particle size is smaller than 250 μm, confirming that internal diffusion is not a factor under the given reaction conditions.

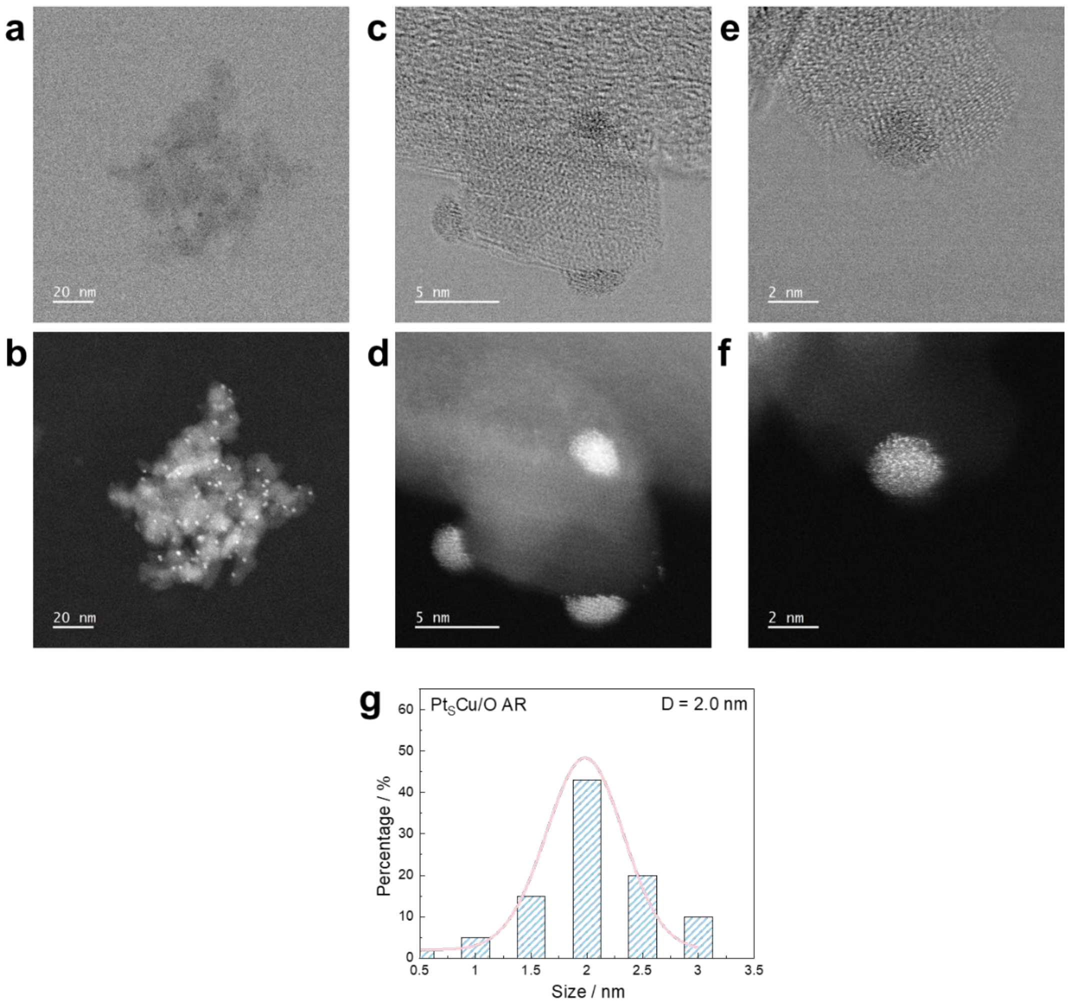

Figure S11. STEM images and particle size distribution of Pt_sCuO/Al_2O_3 after reaction. BF-STEM (**a, c, e**) and HAADF-STEM (**b, d, f**) images of Pt_SCuO/Al_2O_3 after reaction at different magnifications and the particle size distribution (**g**). The average particle size was calculated based on more than 100 particles.

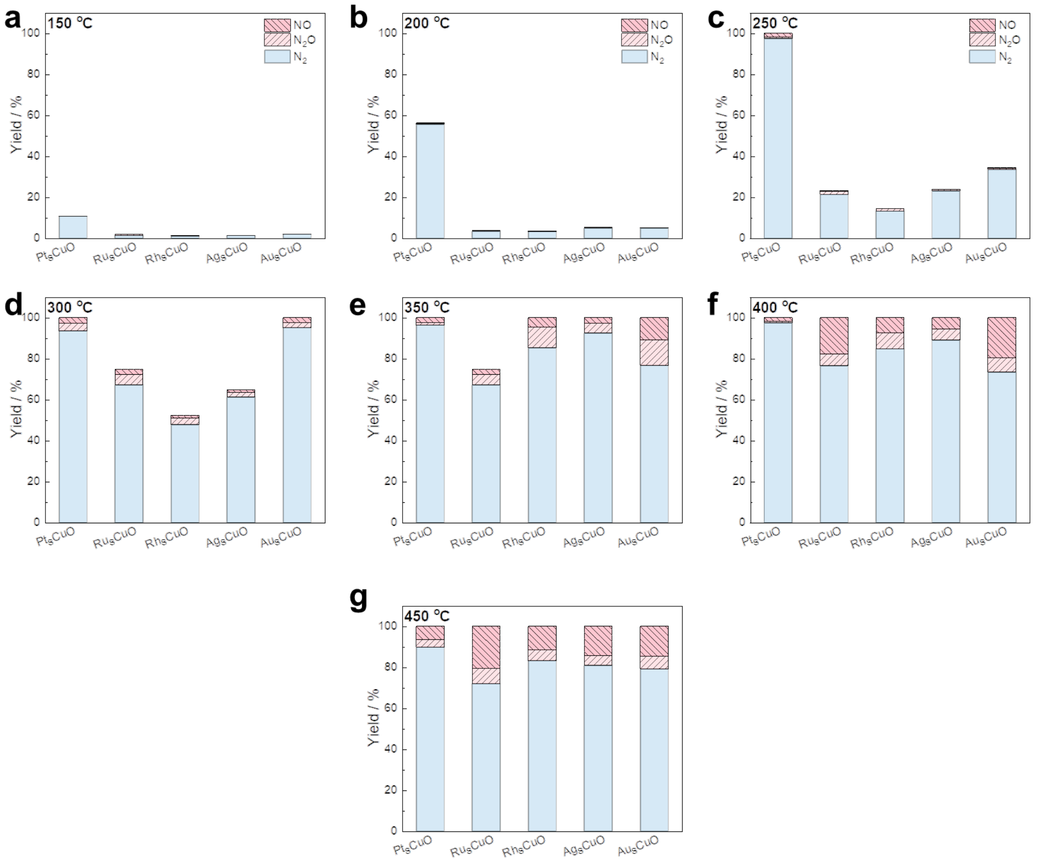

Figure S12. Catalytic performance of M-Cu catalysts (Pt_SCuO/Al_2O_3, Ru_SCuO/Al_2O_3, Rh_SCuO/Al_2O_3, Ag_SCuO/Al_2O_3, Au_SCuO/Al_2O_3) in NH_3 oxidation at different temperatures. a 150 ℃, **b** 200 ℃, **c** 250 ℃, **d** 300 ℃, **e** 350 ℃, **f** 400 ℃, **g** 450 ℃. Reaction condition: 50 mg catalyst, 5000 ppm NH_3, 5% O_2 balanced in He, gas flow: 100 mL/min, WHSV=600 mL $NH_3 \cdot h^{-1} \cdot g^{-1}$.

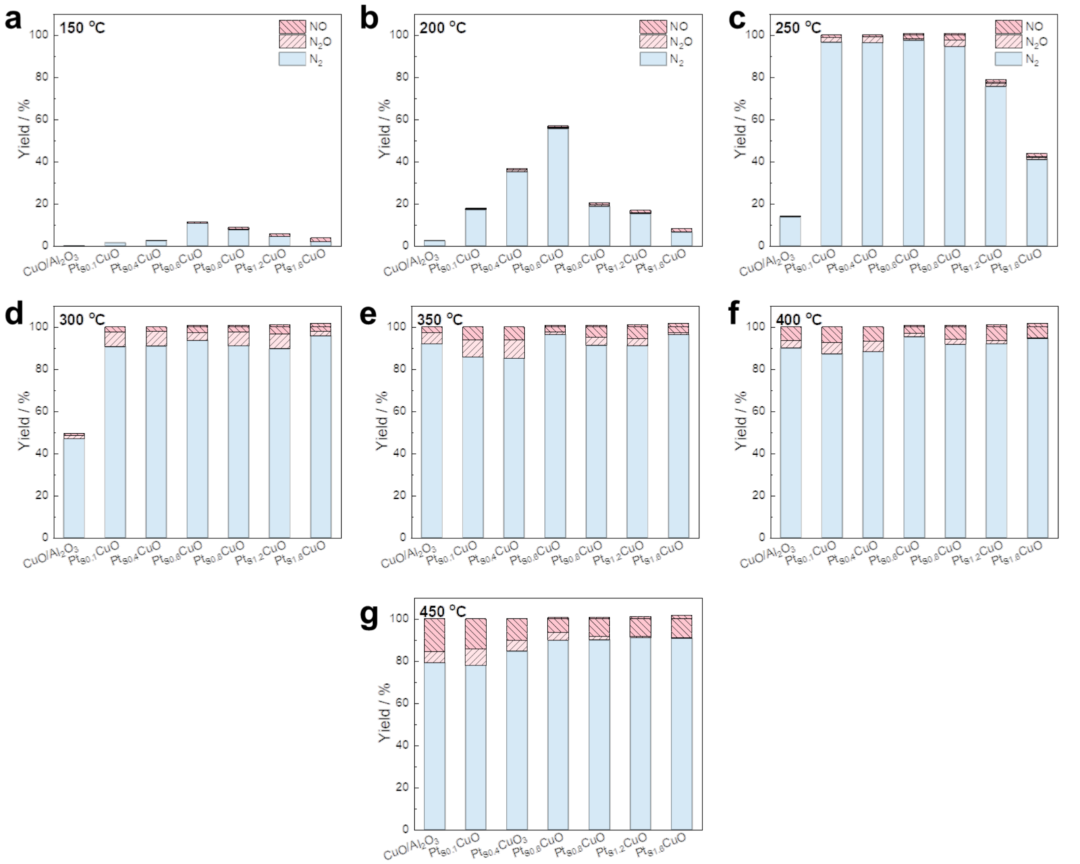

Figure S13. Performance of catalysts with different Pt loadings (0, 0.1, 0.4, 0.6, 0.8, 1.2 and 1.6%) in the oxidation of NH_3 at different temperatures. a 150 °C, **b** 200 °C, **c** 250 °C, **d** 300 °C, **e** 350 °C, **f** 400 °C, **g** 450 °C. Reaction conditions: 50 mg catalyst, 5000 ppm NH_3, 5% O_2 balanced in He, gas flow: 100 mL/min, WHSV=600 mL $NH_3 \cdot h^{-1} \cdot g^{-1}$.

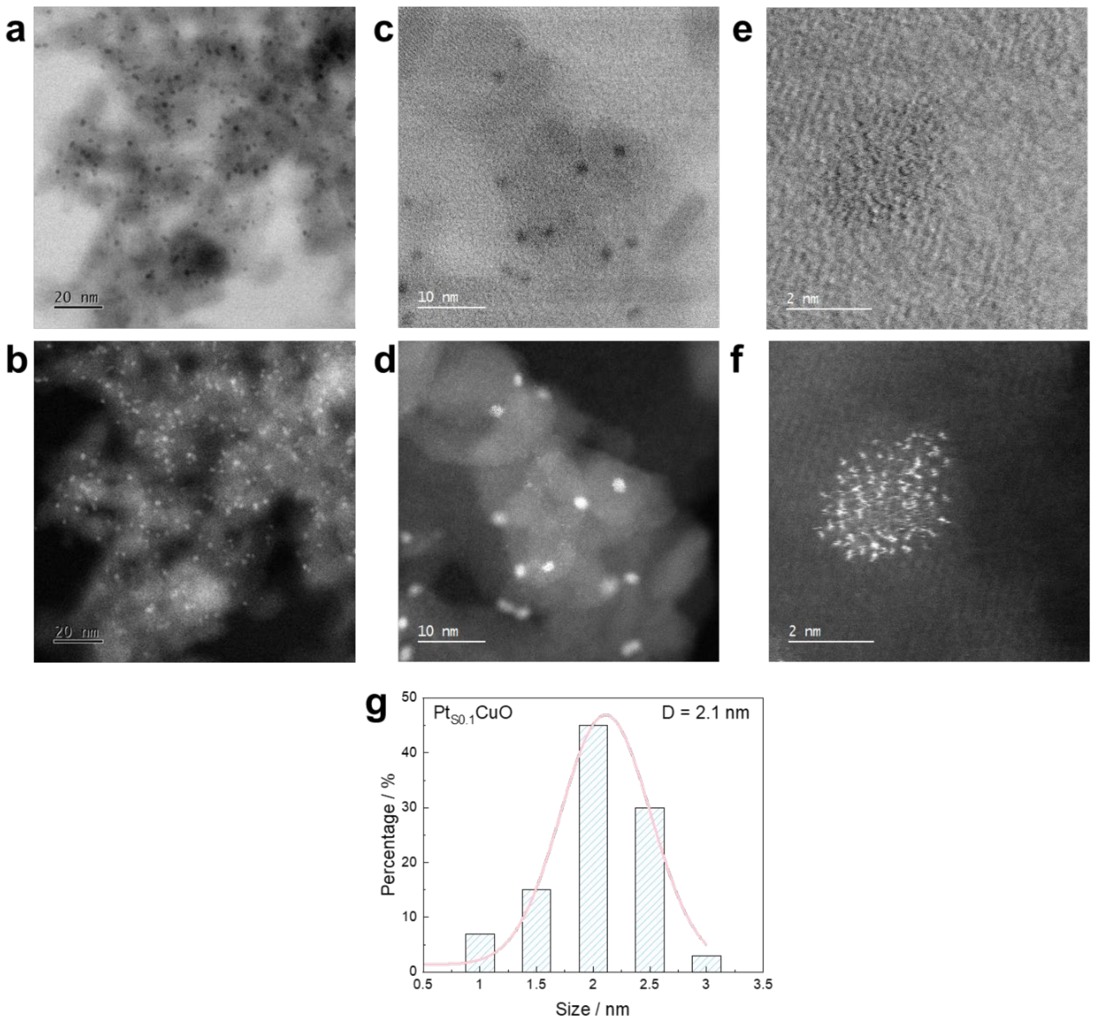

Figure S14. STEM images and particle size distribution of $Pt_{S0.1}CuO/Al_2O_3$. BF-STEM (**a, c, e**) and HAADF-STEM (**b, d, f**) images of $Pt_{S0.1}CuO/Al_2O_3$ at different magnifications and the particle size distribution (**g**). The average particle size was calculated based on more than 100 particles.

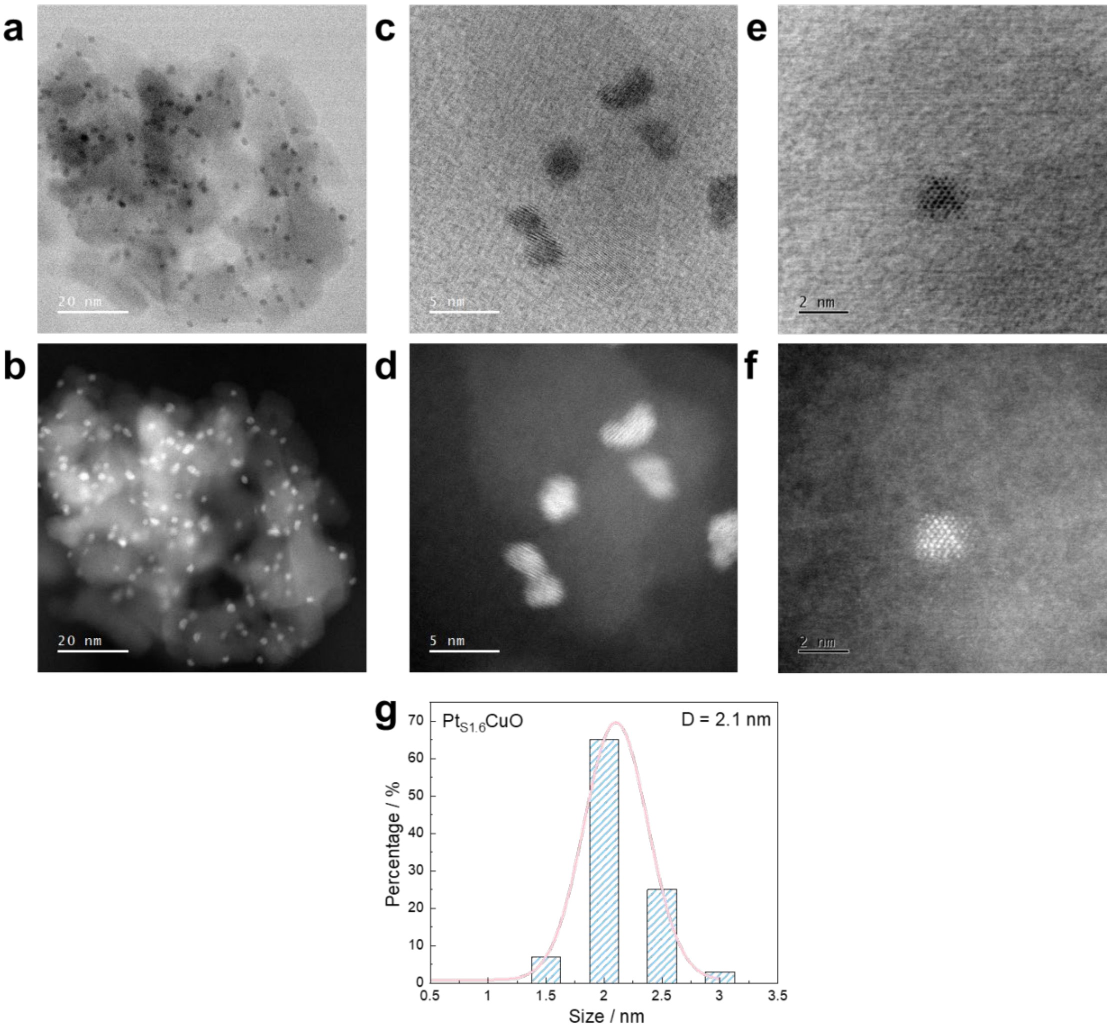

Figure S15. STEM images and particle size distribution of Pt$_{S1.6}$CuO/Al_2O_3 before reaction. BF-STEM (**a, c, e**) and HAADF-STEM (**b, d, f**) images of Pt$_{S1.6}$CuO/Al_2O_3 at different magnifications and the particle size distribution (**g**). The average particle size was calculated based on more than 100 particles.

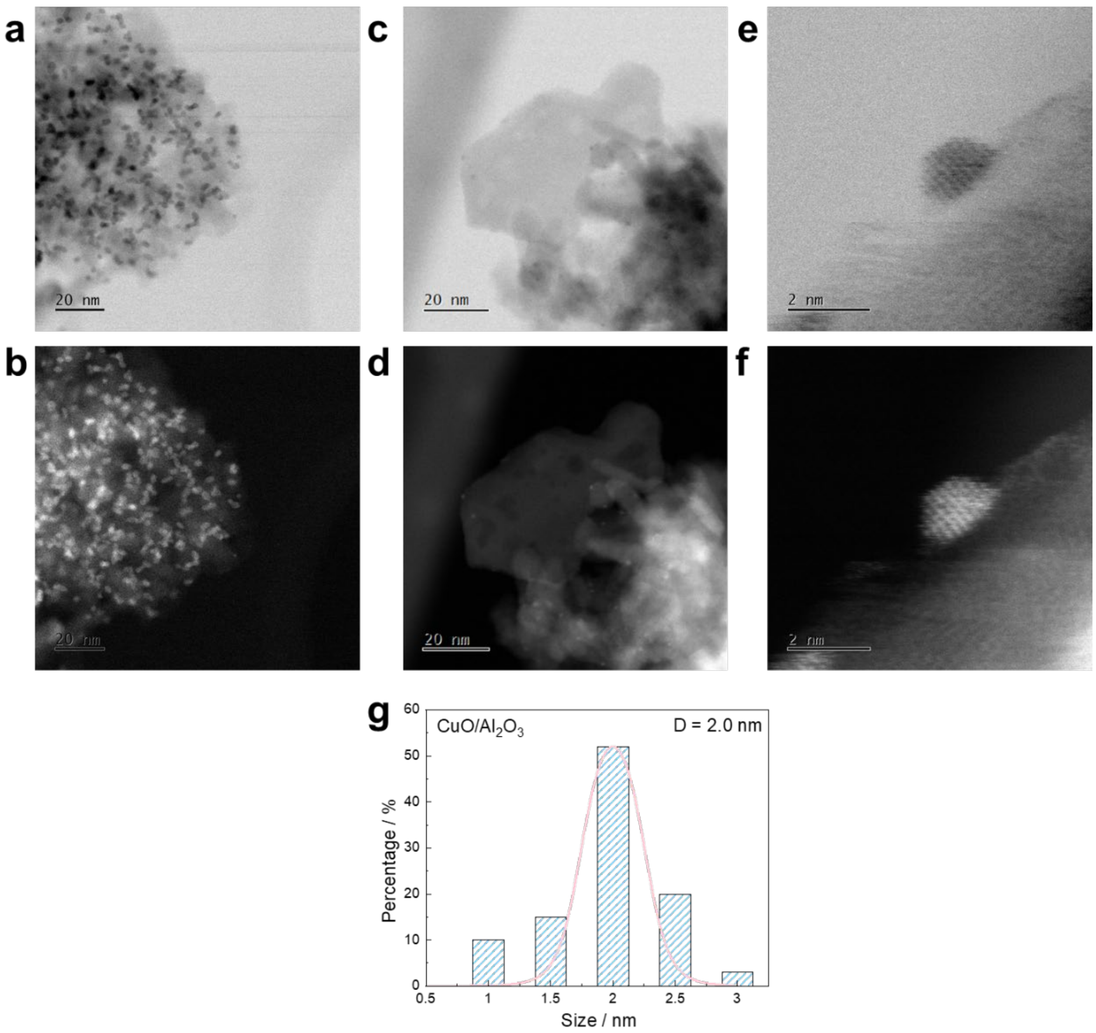

Figure S16. STEM images and particle size distribution of CuO/Al_2O_3. BF-STEM (**a, c, e**) and HAADF-STEM (**b, d, f**) images of CuO/Al_2O_3 at different magnifications and the particle size distribution (**g**). The average particle size was calculated based on more than 100 particles.

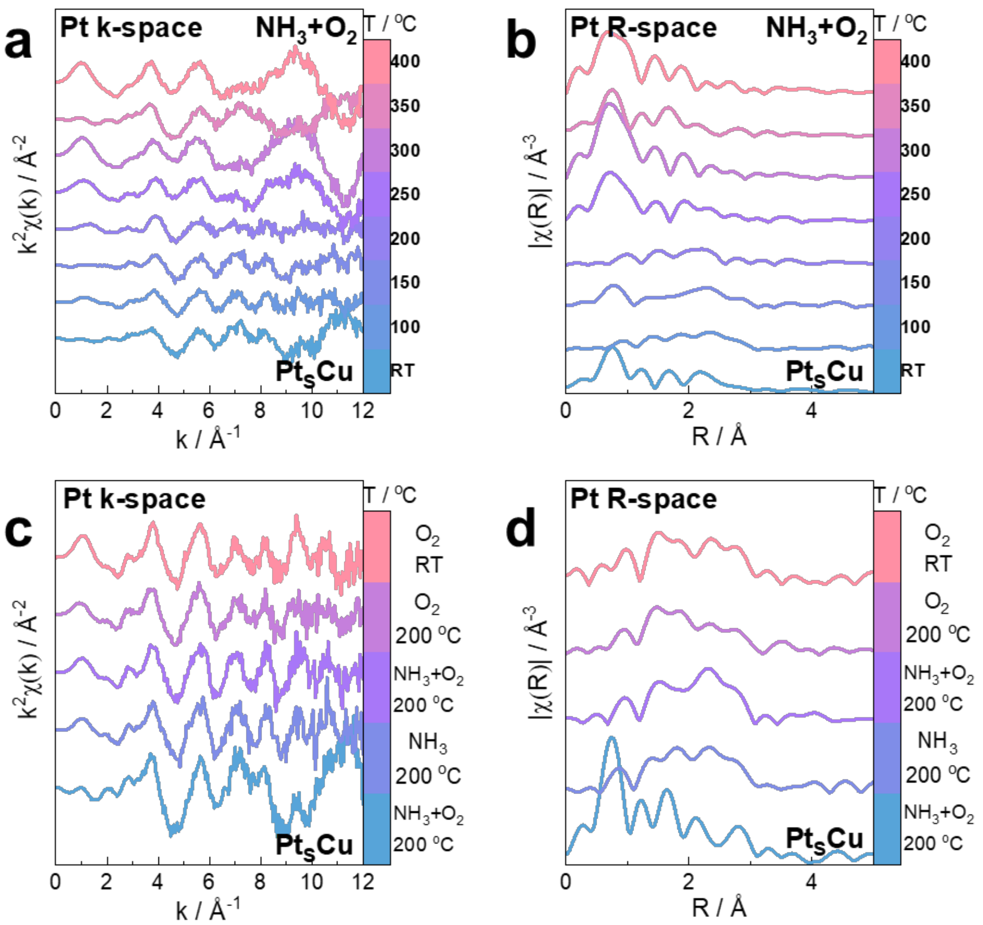

Figure S17. *Operando* Pt L_3-edge EXAFS spectra of Pt_5CuO/Al_2O_3 at different reaction temperatures and in different gases. *Operando* EXAFS in k-space (**a**) and in R-space (**b**) of the Pt L_3-edge of Pt_5CuO/Al_2O_3 as a function of temperature; *Operando* EXAFS in k-space (**c**) and in R-space (**d**) of the Pt L_3-edge of Pt_5CuO/Al_2O_3 in different gas environments.

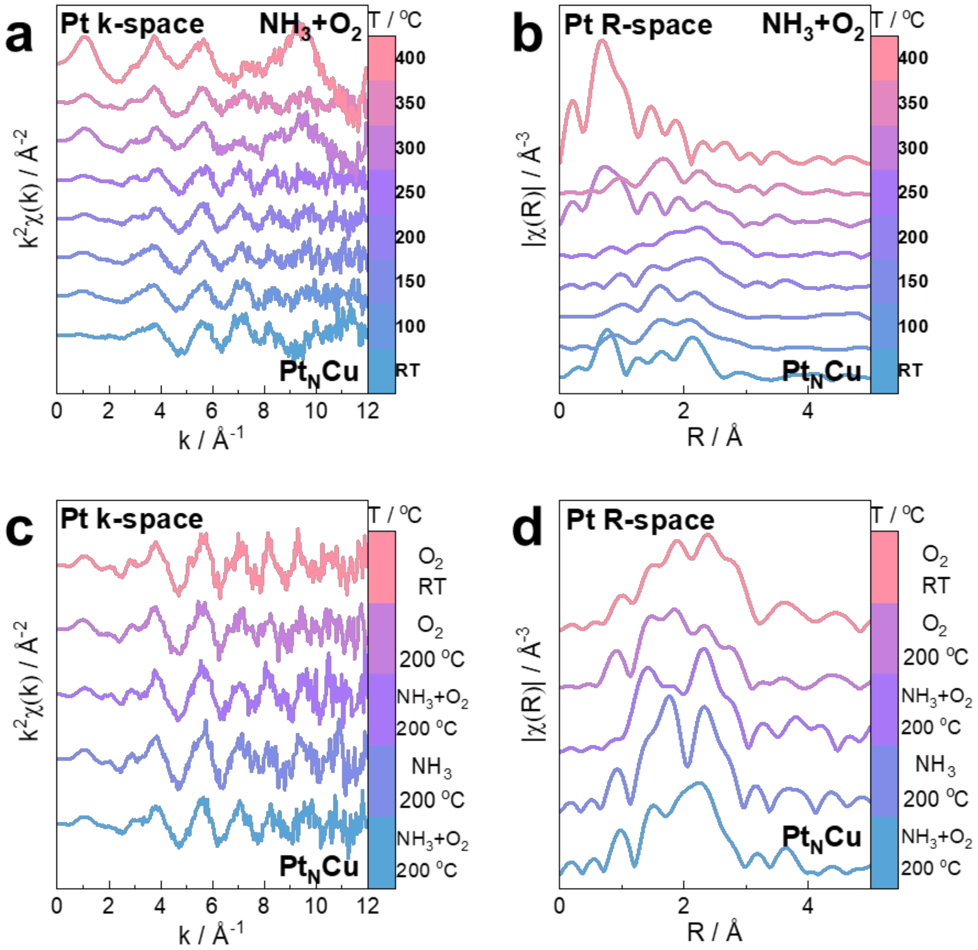

Figure S18. ***Operando*** **Pt L_3-edge EXAFS spectra of Pt_NCuO/Al_2O_3 at different reaction temperatures and in different gases.** *Operando* EXAFS in k-space (**a**) and in R-space (**b**) of the Pt L_3-edge of Pt_NCuO/Al_2O_3 as a function of temperature; *Operando* EXAFS in k-space (**c**) and in R-space (**d**) of the Pt L_3-edge of Pt_NCuO/Al_2O_3 in different gas environments.

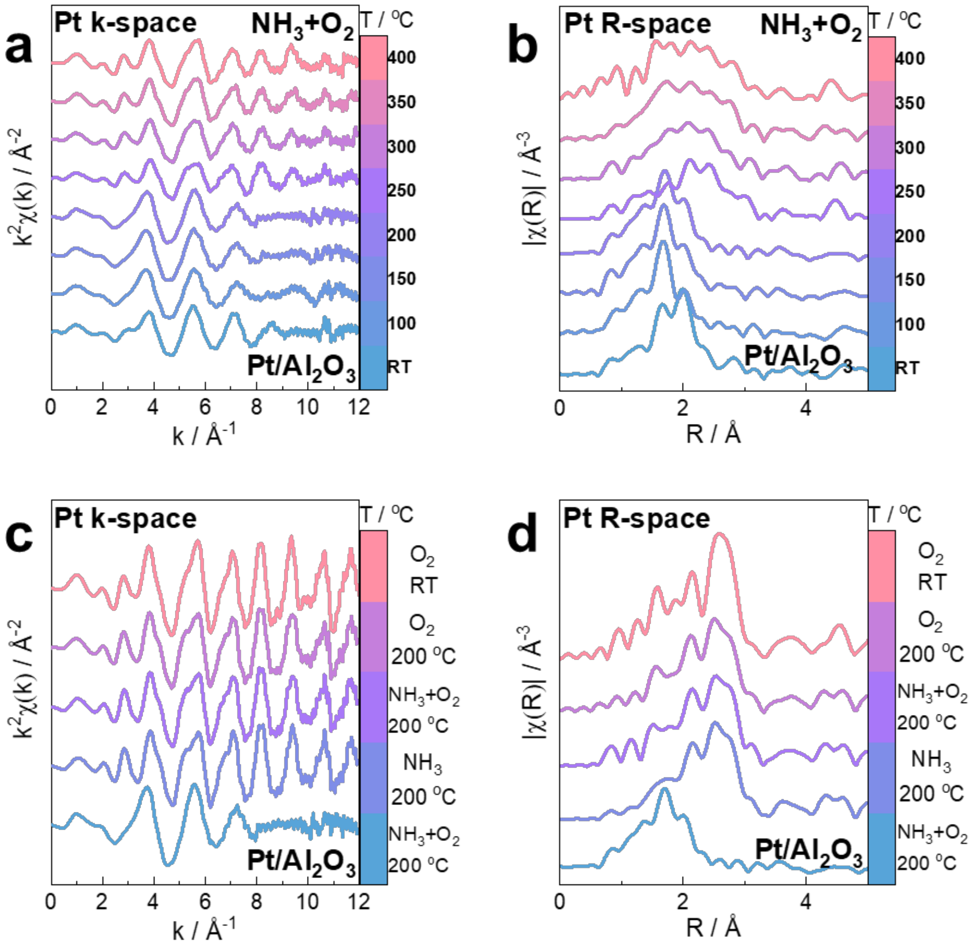

Figure S19. *Operando* Pt L_3-edge EXAFS spectra of Pt/Al_2O_3 at different reaction temperatures and in different gases. *Operando* EXAFS in k-space (**a**) and in R-space (**b**) of the Pt L_3-edge of Pt/Al_2O_3 as a function of temperature; *Operando* EXAFS in k-space (**c**) and in R-space (**d**) of the Pt L_3-edge of Pt/Al_2O_3 in different gas environments.

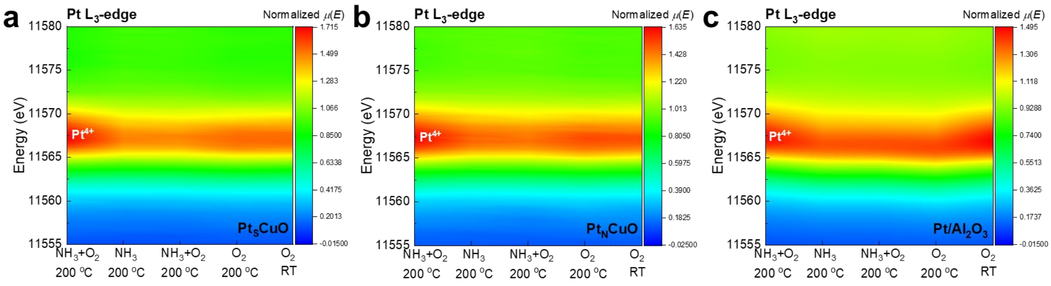

Figure S20. *Operando* Pt L_3-edge XANES spectra of different catalysts in different gases. *Operando* Pt L_3-edge XANES spectra of Pt_SCuO/Al_2O_3 (**a**), Pt_NCuO/Al_2O_3 (**b**) and CuO/Al_2O_3 (**c**) under different gas atmospheres.

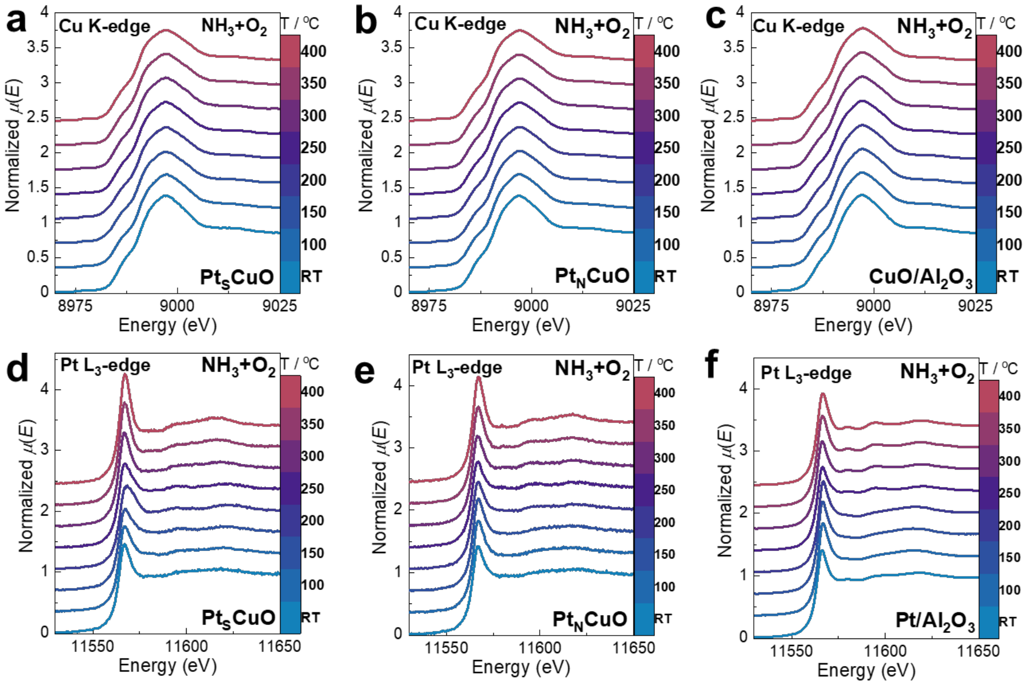

Figure S21. *Operando* Cu K-edge XANES spectra at different reaction temperatures. *Operando* Cu K-edge XANES spectra of Pt_SCuO/Al_2O_3 (**a**), Pt_NCuO/Al_2O_3 (**b**) and CuO/Al_2O_3 (**c**) as a function of temperature; *Operando* Pt L_3-edge XANES spectra of Pt_SCuO/Al_2O_3 (**d**), Pt_NCuO/Al_2O_3 (**e**) and Pt/Al_2O_3 (**f**) as a function of temperature.

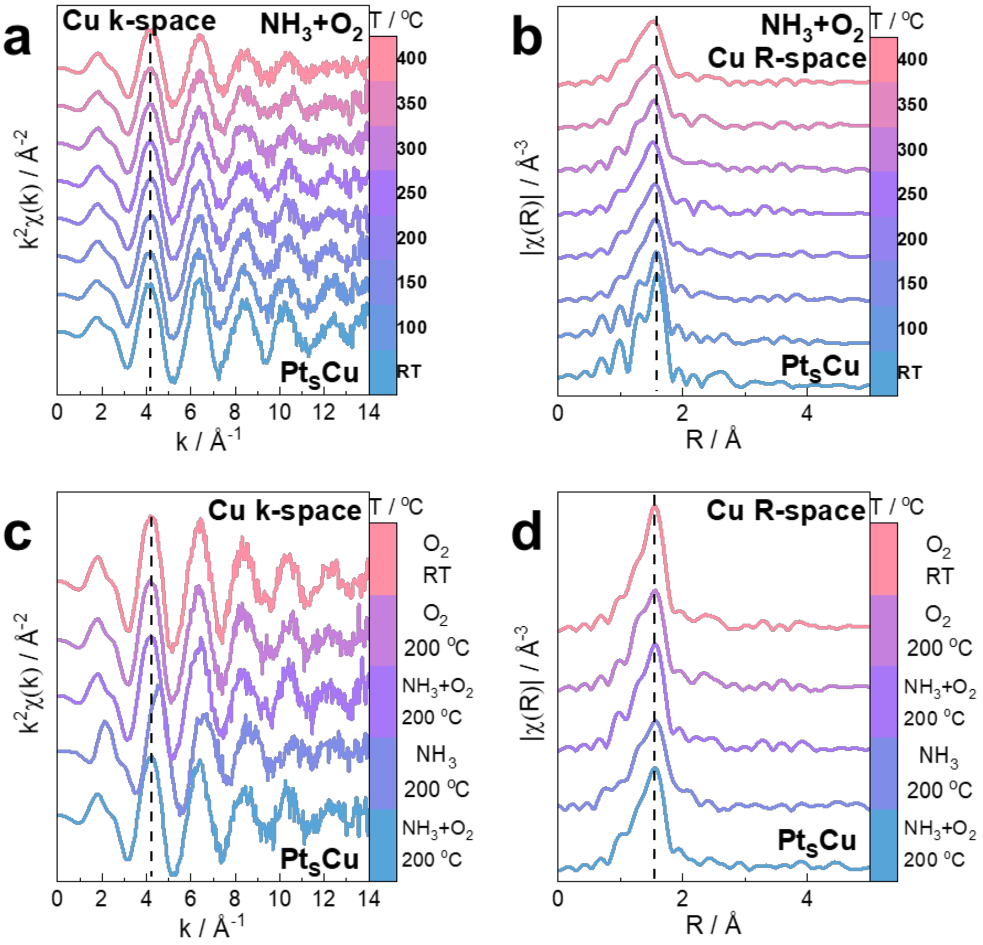

Figure S22. ***Operando*** **Cu K-edge EXAFS spectra of Pt_SCuO/Al_2O_3 at different reaction temperatures and in different gases.** *Operando* EXAFS in k-space (**a**) and in R-space (**b**) of the Cu K-edge of Pt_SCuO/Al_2O_3 as a function of temperature; *Operando* EXAFS in k-space (**c**) and in R-space (**d**) of the Cu K-edge of Pt_SCuO/Al_2O_3 in different gas environments.

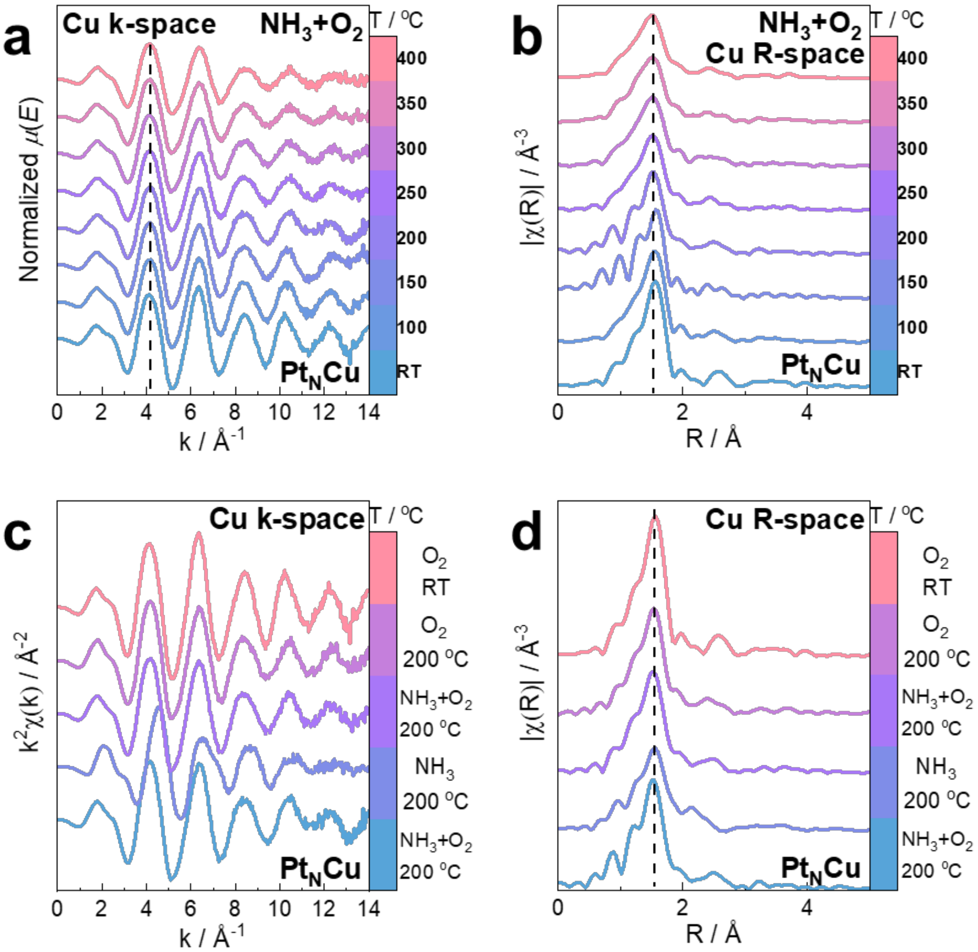

Figure S23. ***Operando*** **Cu K-edge EXAFS spectra of Pt_NCuO/Al_2O_3 at different reaction temperatures and in different gases.** *Operando* EXAFS in k-space (**a**) and in R-space (**b**) of the Cu K-edge of Pt_NCuO/Al_2O_3 as a function of temperature; *Operando* EXAFS in k-space (**c**) and in R-space (**d**) of the Cu K-edge of Pt_NCuO/Al_2O_3 in different gas environments.

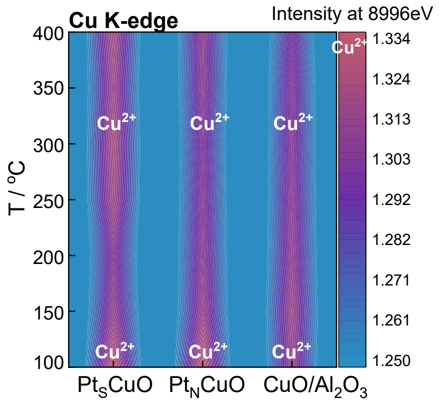

Figure S24. ***Operando*** **Cu K-edge XAFS at different reaction temperatures.** Signal intensity of the white-line at 8996 eV in a NH_3/O_2 atmosphere at different temperatures.

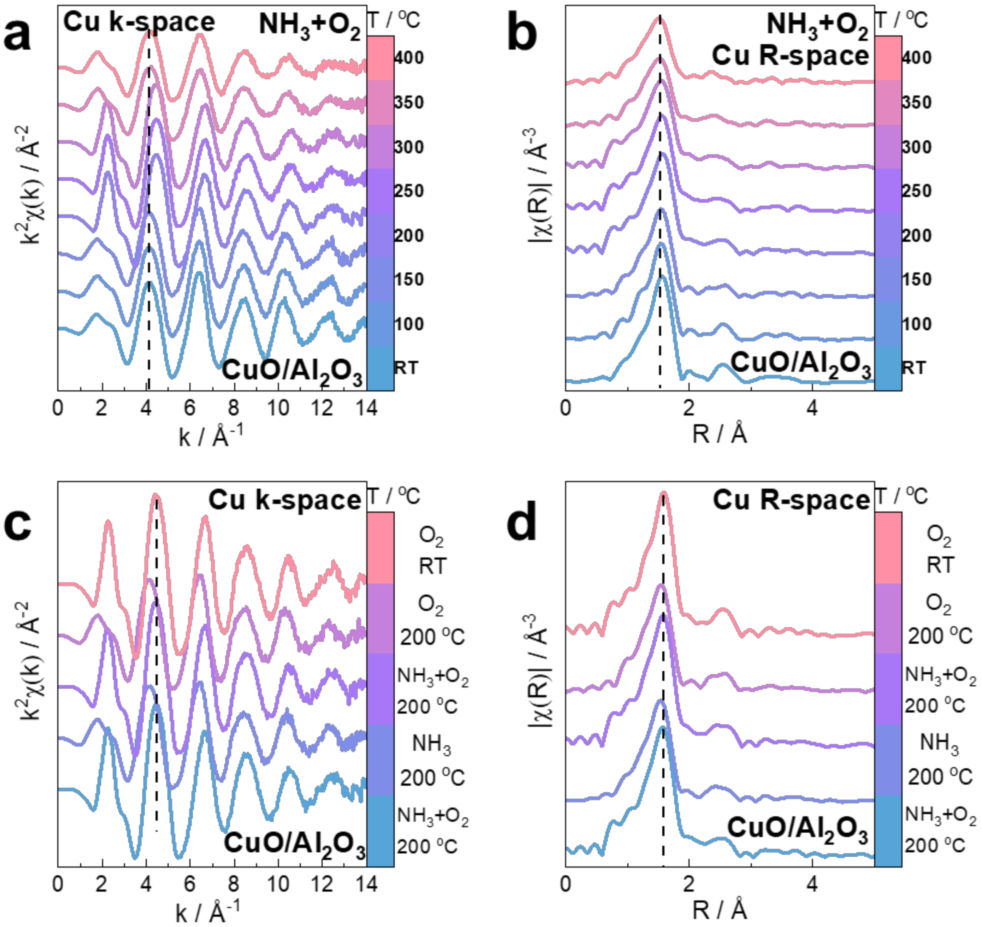

Figure S25. ***Operando*** **Cu K-edge EXAFS spectra of CuO/Al_2O_3 at different reaction temperatures and in different gases.** *Operando* EXAFS in k-space (**a**) and in R-space (**b**) of the Cu K-edge of CuO/Al_2O_3 as a function of temperature; *Operando* EXAFS in k-space (**c**) and in R-space (**d**) of the Cu K-edge of CuO/Al_2O_3 in different gas environments.

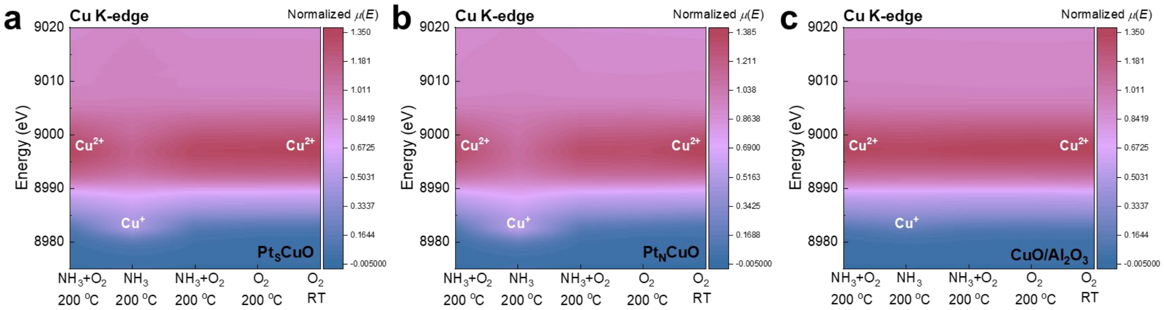

Figure S26. ***Operando*** **Cu K-edge XANES spectra of different catalysts under different gases.** *Operando* Cu K-edge XANES spectra of Pt_SCuO/Al_2O_3 (**a**), Pt_NCuO/Al_2O_3 (**b**) and CuO/Al_2O_3 (**c**) under different gas atmospheres.

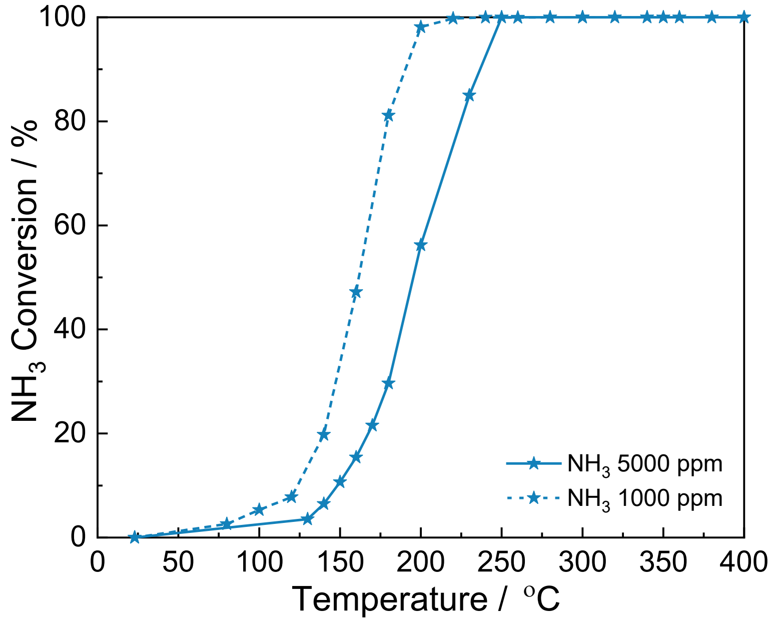

Figure S27. Light off curve for Pt_5Cu/Al_2O_3 at different NH_3 concentration. Reaction conditions: 50 mg catalyst, 1000 or 5000 ppm NH_3, 5% O_2 balanced in He, gas flow: 100 mL/min.

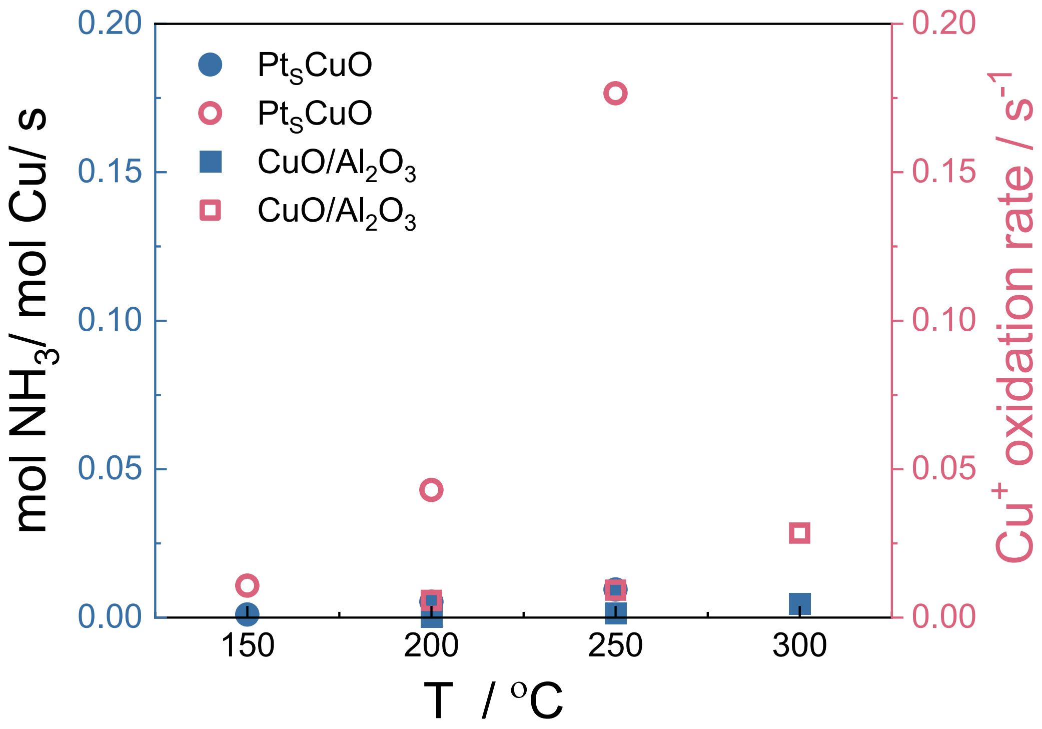

Figure S28. Turnover frequency (TOF) comparations between Pt_SCu/Al_2O_3 and CuO/Al_2O_3. TOF of the whole NH_3-SCO reaction (i.e. NH_3 oxidation rate) and Cu^+ oxidation rate in Pt_SCu/Al_2O_3 and CuO/Al_2O_3

Supplementary Tables

Table S1. EXAFS fitting results of the different catalysts.

Sample	Scattering	C.N.	d (Å)	σ^2	E_0 (eV)	R-factor
Cu foil STD	Cu-Cu	12	2.56			
Cu_2O STD	Cu-O	2	1.85			
	Cu-Cu	12	3.01			
CuO STD	Cu-O	4	1.95			
	Cu-Cu	4<br>4<br>2	2.88<br>3.07<br>3.16			
Pt_SCuO/Al_2O_3	Cu-O	3.42± 0.17	1.95	0.005± 0.0007	-0.39±0.70	0.013
	Pt-O (1)	2.01 ± 0.55	1.95	0.006±0.004	1.85± 2.30	0.017
	Pt-Pt (2)	5.77 ± 1.46	2.69	0.009±0.002		
	Pt-Cu (3)	1.08 ± 0.66	2.67			
Pt_NCuO/Al_2O_3	Cu-O	3.41± 0.16	1.95	0.003±0.0007	-0.61±0.62	0.015
	Pt-O (1)	1.75± 0.32	1.91	0.005±0.0001	2.10± 2.80	0.022
	Pt-Pt (2)	4.48± 1.36	2.79	0.011±0.003		
	Pt-Cu (3)	5.84± 1.72	2.56			

a C.N. is coordination number.
b d is interatomic distance.
c σ^2 is Debye-Waller factor, a measure of thermal and static disorder in absorber scatter distances.
d $\Delta E0$ is edge energy shift.
EXAFS data fitting was in accordance with literature methods.[1–3]

Table S2. Review of performance of CuO-based bi-functional catalysts evaluated in the NH_3-SCO reaction.

No.	Catalyst	T (°C)	NH_3 conversion (%)	N_2 selectivity (%)	WHSV ($mL_{NH3}\cdot h^{-1}\cdot g^{-1}$)	Ref.
1	Pt_SCuO/Al_2O_3 (this work)	200	100	96	600	
2		400	100	98	600	
3	10wt%CuO-Al_2O_3	350	100	93	30	4
4	Pt/Al_2O_3 (1.8 wt.% Pt)	220	100	63	206	5
5	$Pt/CuO/Al_2O_3$ (1.0 wt.% Pt, 20.0 wt.% Cu)	210	100	88	126	6
6	$Pt/CuO/Al_2O_3$ (1.0 wt.% Pt, 20.0 wt.% Cu)	250	100	83	-	7
7	$Au/Cu/Al_2O_3$ (5 wt.% Au/1.0 mol% Cu)	300	100	95	240	8
8	$Ag/Cu/Al_2O_3$ (7.5 wt.% Ag/2.5 wt.% Cu)	300	100	95	30	9
9	$Ag/Cu/Al_2O_3$ (7.5 wt.% Ag/2.5 wt.% Cu)	325	100	95	300	10
10	Pt/Al_2O_3-Cu/ZSM-5 (0.46 wt.% Pt/2.5 wt.% Cu)	250	100	82	-	11
11	30wt%CuO-RuO_2	210	100	99	75	12
12	4.2 wt% Pd/Al_2O_3	300	100	86	300	13
13	2.6 wt% Pt/Al_2O_3	250	98	43	299	13
14	Pt/Al_2O_3@Cu/ZSM-5	300	98	94	167	14
15	Pt/Al_2O_3 + Cu/SSZ-13	450	90	80	524	15
16	Pt/Al_2O_3 + Cu/SSZ-13	450	80	90	GHSV: 332k h^{-1}	16
17	2 wt % Pt/γ-Al_2O_3	230	100	40	1260	17

Supplementary References

1. Feiten, F. E. *et al.* Model building analysis-a novel method for statistical evaluation of Pt L3-edge EXAFS data to unravel the structure of Pt-alloy nanoparticles for the oxygen reduction reaction on highly oriented pyrolytic graphite. *Phys. Chem. Chem. Phys.* **22**, 18815–18823 (2020).
2. Pryadchenko, V. V. *et al.* Bimetallic PtCu core-shell nanoparticles in PtCu/C electrocatalysts: Structural and electrochemical characterization. *Appl. Catal. A Gen.* **525**, 226–236 (2016).
3. Srabionyan, V. V. *et al.* Atomic structure of PtCu nanoparticles in PtCu/C catalysts from EXAFS spectroscopy data. *Phys. Solid State* **58**, 752–762 (2016).
4. Liang, C., Li, X., Qu, Z., Tade, M. & Liu, S. The role of copper species on Cu/γ-Al2O3 catalysts for NH3–SCO reaction. *Appl. Surf. Sci.* **258**, 3738–3743 (2012).
5. Svintsitskiy, D. A. *et al.* The State of Platinum and Structural Features of Pt/Al2O3 Catalysts in the Reaction of NH3 Oxidation. *J. Struct. Chem.* **60**, 919–931 (2019).
6. Olofsson, G., REINEWALLENBERG, L., ANDERSSON, A., Wallenberg, L. R. & ANDERSSON, A. Selective catalytic oxidation of ammonia to nitrogen at low temperature on Pt/CuO/Al2O3. *J. Catal.* **230**, 1–13 (2005).
7. Kušar, H. M. J., Ersson, A. G., Vosecký, M. & Järås, S. G. Selective catalytic oxidation of NH3 to N2 for catalytic combustion of low heating value gas under lean/rich conditions. *Appl. Catal. B Environ.* **58**, 25–32 (2005).
8. Lin, S. D., Gluhoi, A. C. & Nieuwenhuys, B. E. Ammonia oxidation over Au/MO /γ-Al2O3—activity, selectivity and FTIR measurements. *Catal. Today* **90**, 3–14 (2004).
9. Gang, L. *et al.* Alumina-Supported Cu–Ag Catalysts for Ammonia Oxidation to Nitrogen at Low Temperature. *J. Catal.* **206**, 60–70 (2002).
10. Yang, M., Wu, C., Zhang, C. & He, H. Selective oxidation of ammonia over copper-silver-based catalysts. *Catal. Today* **90**, 263–267 (2004).
11. Shrestha, S., Harold, M. P., Kamasamudram, K. & Yezerets, A. Ammonia Oxidation on Structured Composite Catalysts. *Top. Catal.* **56**, 182–186 (2013).
12. Cui, X. *et al.* Selective catalytic oxidation of ammonia to nitrogen over mesoporous CuO / RuO 2 synthesized by co-nanocasting-replication method. *J. Catal.* **270**, 310–317 (2010).
13. Li, Y. & Armor, J. N. Selective NH3 oxidation to N2 in a wet stream. *Appl. Catal. B Environ.* **13**, 131–139 (1997).
14. Ghosh, R. S. *et al.* Enhanced Selective Oxidation of Ammonia in a Pt/Al2O3@Cu/ZSM-5 Core-Shell Catalyst. *ACS Catal.* **10**, 3604–3617 (2020).
15. Dhillon, P. S., Harold, M. P., Wang, D., Kumar, A. & Joshi, S. Y. Optimizing the dual-layer Pt/Al2O3 + Cu/SSZ-13 washcoated monolith: Selective oxidation of NH3 to N2. *Catal. Today* **360**, 426–434 (2021).
16. Dhillon, P. S., Harold, M. P., Wang, D., Kumar, A. & Joshi, S. Y. Modeling and analysis of transport and reaction in washcoated monoliths: Cu-SSZ-13 SCR and dual-layer Cu-SSZ-13 + Pt/Al2O3 ASC. *React. Chem. Eng.* **4**, 1103–1115 (2019).
17. Marchuk, V., Sharapa, D. I., Grunwaldt, J. D. & Doronkin, D. E. Surface States Governing the Activity and Selectivity of Pt-Based Ammonia Slip Catalysts for Selective Ammonia Oxidation. *ACS Catal.* **14**, 1107–1120 (2024).
